# A Review on Recent Advances in Nitrogen-Containing Molecules and Their Biological Applications

**DOI:** 10.3390/molecules25081909

**Published:** 2020-04-20

**Authors:** Nagaraju Kerru, Lalitha Gummidi, Suresh Maddila, Kranthi Kumar Gangu, Sreekantha B. Jonnalagadda

**Affiliations:** School of Chemistry & Physics, University of KwaZulu-Natal, Westville Campus, Chiltern Hills, Durban 4000, South Africa; nagarajukerru@gmail.com (N.K.); gummidilalitha@gmail.com (L.G.); sureshmskt@gmail.com (S.M.); kkgangu@ymail.com (K.K.G.)

**Keywords:** nitrogen-based heterocycles, current trends, biological activities, structure-activity relationship

## Abstract

The analogs of nitrogen-based heterocycles occupy an exclusive position as a valuable source of therapeutic agents in medicinal chemistry. More than 75% of drugs approved by the FDA and currently available in the market are nitrogen-containing heterocyclic moieties. In the forthcoming decade, a much greater share of new nitrogen-based pharmaceuticals is anticipated. Many new nitrogen-based heterocycles have been designed. The number of novel *N*-heterocyclic moieties with significant physiological properties and promising applications in medicinal chemistry is ever-growing. In this review, we consolidate the recent advances on novel nitrogen-containing heterocycles and their distinct biological activities, reported over the past one year (2019 to early 2020). This review highlights the trends in the use of nitrogen-based moieties in drug design and the development of different potent and competent candidates against various diseases.

## 1. Introduction

Nitrogen-based heterocyclic chemistry is an important and unique class among the applied branches of organic chemistry, with a significant amount of research dedicated to the development of novel molecules and composites. These molecules have received increasing attention over the past two decades. They contributed to the development of numerous organic synthesis protocols and found abundant applications in the chemical sciences [1,2,3,4,5]. Many *N*-heterocyclic compounds that are broadly distributed in Nature, possess physiological and pharmacological properties and are constituents of many biologically important molecules, including many vitamins, nucleic acids, pharmaceuticals, antibiotics, dyes and agrochemicals, amongst many others [6,7,8,9,10]. Moreover, they form an integral part of many pharmacologically active molecules. The base pairs of DNA and RNA (guanine, cytosine, adenine, and thymine) are also made up of *N*-heterocyclic compounds, namely purines, pyrimidines, etc. These nitrogen-containing heterocyclic molecules with distinct characteristics and applications have gained prominence in the rapidly expanding fields of organic and medicinal chemistry and the pharmaceutical industry [11,12,13]. Furthermore, the electron-rich nitrogen heterocycle is not only able to readily accept or donate a proton, but it can also easily establish diverse weak interactions. Some of these intermolecular forces, such as like hydrogen bonding formation, dipole-dipole interactions, hydrophobic effects, van der Waals forces and π-stacking interactions of nitrogen compounds have increased their importance in the field of medicinal chemistry and allows them to bind with a variety of enzymes and receptors in biological targets with high affinity due to their improved solubility. The structural features of their derivatives are beneficial sice they exhibit broad bioactivities.

**Figure 1:**
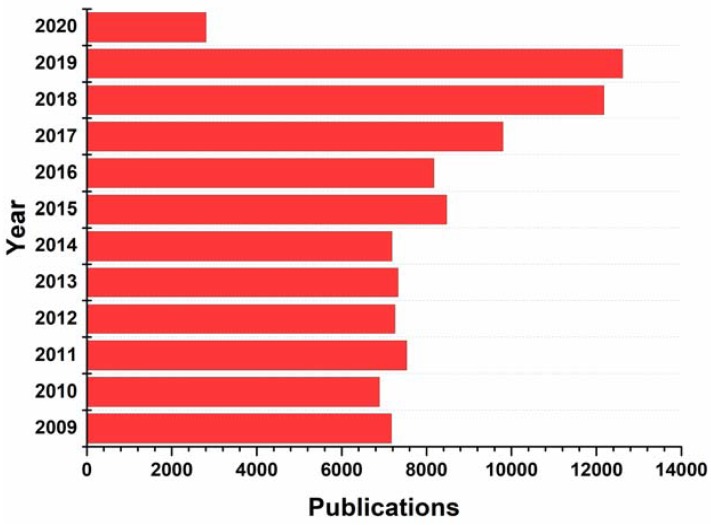
Publications on nitrogen-based heterocycles between 2009 to early 2020 (Source Scopus) [26].

A glance at the FDA databases reveals the structural significance of nitrogen-based heterocycles in drug design and engineering of pharmaceuticals. Nearly 75% unique small-molecule drugs contain a nitrogen heterocycle. Recently, Vitaku and colleagues published a comprehensive compilation of the structural diversity, substitution patterns and frequency of nitrogen heterocycles among U.S. FDA approved pharmaceuticals [14]. The *N*-heterocyclic skeletons feature significantly various classes of therapeutic applications and are used as the building blocks of a number of new drug candidates, due to the ability of the nitrogen atom to easily form hydrogen bonding with biological targets [15,16,17]. For example, pyrimidine derivatives have various therapeutic applications in medicinal chemistry and pyrimidine skeleton of thymine, cytosine, and uracil are essential building blocks of nucleic acids, DNA and RNA [18]. A vast number of nitrogen-containing heterocyclic compounds are known to exhibit a wide range of pharmacological activities including anticancer, anti-HIV, antimalaria, anti-tubercular, anti-microbial and diabetic activities [19,20,21,22,23,24,25]. A measure of their importance in the design of nitrogen heterocycles can be seen by the over 97,400 publications on nitrogen heterocycles that have appeared between 2009 and early 2020, as illustrated in Figure 1, of which 12,615 publications have been reported in the year 2019 [26]. Particularly, between the years 282 publications have been reported with medicinal chemistry related *N*-heterocycles. The utility of *N*-heterocyclic compounds is a hot spot in medicinal chemistry and chemical science, as can be seen by the list of nitrogen-containing clinical drugs (Table 1). The exceptional role of nitrogen for variable interactions with biological targets, broadens the scope for drug development.

This review abstracts information available over the past year (2019–2020) to provide an insight into the current status of nitrogen-containing heterocyclic molecules and their biological profile. The review evaluates the recent progress on different *N*-bearing heterocyclic structures (four-, five- and six-membered rings) introduced (i.e., β-lactams, pyrazoles, imidazoles, 1,2,4-triazoles, pyrimidines, quinolines, and quinazolines) and discusses their potential medicinal importance. An appraisal is provided for the rational design of more active molecules. We believe that this article will serve as an informative reference for the medicinally important nitrogen-containing heterocycles.

## 2. Four-Membered Ring Heterocycles

The nitrogen-containing four-membered ring heterocycles have proved their biological importance in medicinal chemistry and have further increased their biological significance.

### β-Lactams

**Figure 2:**
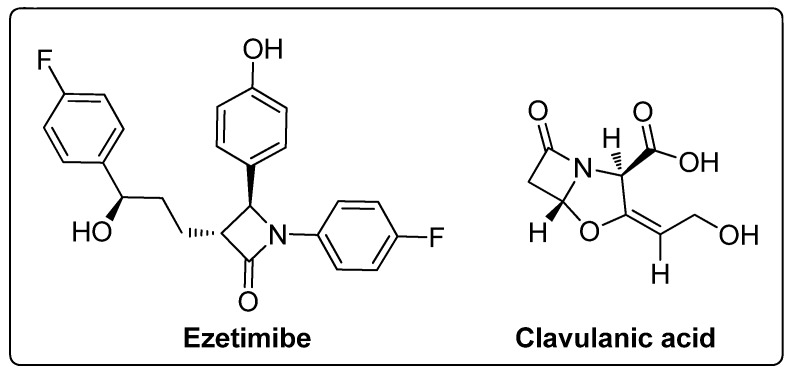
β-Lactam clinical drugs.

Among the heterocyclic compounds, the four-membered cyclic amide ring system of β-lactams has evolved as the scaffold of choice in the design of many antibiotics and it is also a valuable building block in organic synthesis. It is recognized as a vital component for the bioactivity profile of antibiotics [41,42,43,44,45]. A range of conjugates with diverse pharmacological applications can be synthesized by incorporating the β-lactam structure as a key scaffold or utilizing it as an important building block for the synthesis of a variety of bioactive heterocycles [46,47]. Besides antibiotics, β-lactams have other clinical applications, for example, clavulanic acids are β-lactamase inhibitors and ezetimibe is a cholesterol absorption inhibitor (Figure 2) [48,49]. These diverse applications have drawn attention to the further development of the β-lactam ring and several protocols have been devised to synthesize four-membered ring β-lactams. Currently, research in this area has been stimulated due to the development of bacterial resistance to known β-lactam antibiotics.

**Figure 3:**
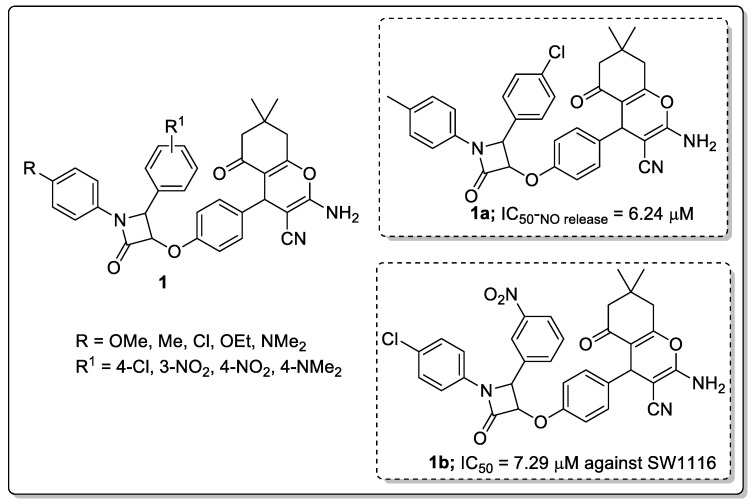
Anti-inflammatory (compound **1a**) and anti-cancer activity (compound **1b**) of the most active chromeno-β-lactam hybrids.

Chromene tagged β-lactam molecular hybrids (**1**) were prepared and evaluated them for their anti-inflammatory and anticancer activities [50]. The authors used the RAW 264.7 murine macrophage assay to examine the anti-inflammatory behavior of the β-lactam hybrids, and evaluated the capacity of the compounds to inhibit the pro-inflammatory cascade, leading to NO production in mouse macrophages. Among the established molecules, compound **1a** with a *para*-methylphenyl moiety at N1 and *para*-chlorophenyl at the C4 position of the β-lactam ring showed the most significant activity with a 19.8 anti-inflammatory ratio (IC_50-NO release_ = 6.24 μM and IC_50-cell viability_ = 123.47 μM) (Figure 3). On the other hand, 4-chlorophenyl at N1 and 3-nitrophenyl at the C4 position of the β-lactam ring hybrid (**1b**) displayed the promising anticancer activity with an IC_50_ value of 7.29 μM (compared to the standard methotrexate (IC_50_ = 2.49 μM)) against the SW1116 colon cancer cell line. Structure-activity relationship (SAR) studies revealed that *p*-methoxy, *p*-tolyl and a *p*-chlorophenyl ring on the *N1* of the β-lactam ring provided enhanced anticancer activity against the colon cancer (SW1116) cell line, whereas, a lower activity was observed against the HepG2 cell line for all the tested compounds. The replacement of the *p*-chlorophenyl group at the C-4 position of the lactam ring with a *p*-nitrophenyl group substantially decreased the in vitro anti-inflammatory activity. This loss of activity may be attributed to the weak π-π interaction between the lactam ring and enzyme active sites as discerned by docking studies. The active compound exhibited more lipophilic activity than other derivatives. Hence, the compound experiences stronger interactions with hydrophobic amino acids, accounting for its better anti-inflammatory activity.

**Figure 4:**
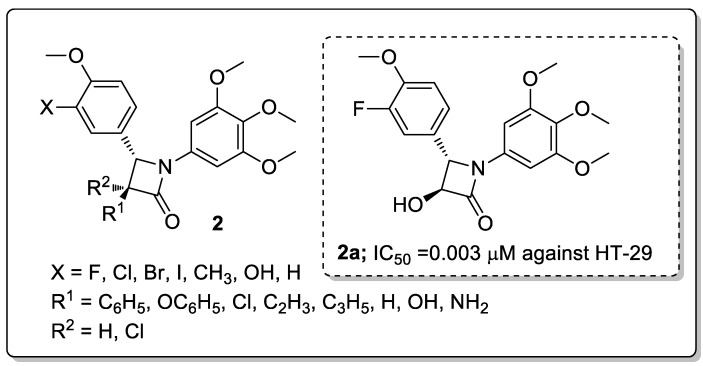
Antiproliferative activity of most potent β-lactam derivative **2a**.

A series of β-lactam analogs **2** were synthesized and screened the analogs for antiproliferative activity against human colon (HT-29) and breast (MCF-7) cancer cell lines [51]. A hydroxy attached at the 3-position of the β-lactam and a fluorophenyl conjugate (compound **2a**) showed excellent antiproliferative activity with IC_50_ values of 0.022 μM against MCF-7 and 0.003 μM against HT-29 cell lines (Figure 4). Reduced activity was observed when the chloro, bromo, and iodo substituents were replaced with a fluorine atom with the following order Br > Cl > I against the colon HT-29 cell line. The introduction of fluorine may improve the pharmacological and physicochemical properties, such as metabolic stability, lipophilicity, and ligand binding of a compound. The active compound, **2a,** induced mitotic arrest (G2/M phase) at a micromolar concentration in both colon and breast cancer cell lines and strongly inhibited the colchicine site of tubulin. The 3,4,5-trimethoxyphenyl group can make favorable van der Waals contacts within the lower subpocket delineated by Valb318 and Cysb241. The overlap of the fluorine atom onto the DAMA-colchicine carbonyl oxygen atom facilitates the HBA interaction with Lysb352, and the hydroxyl group on the β-lactam ring can form an HDB interaction with Lys254. From the molecular modeling study, it can be concluded that the active compound interacts with tubulin at the same site as colchicine with similar binding modes. The β-lactam derivatives are promising candidates in the progress of tubulin-targeting agents for the treatment of colon and breast cancers.

**Figure 5:**
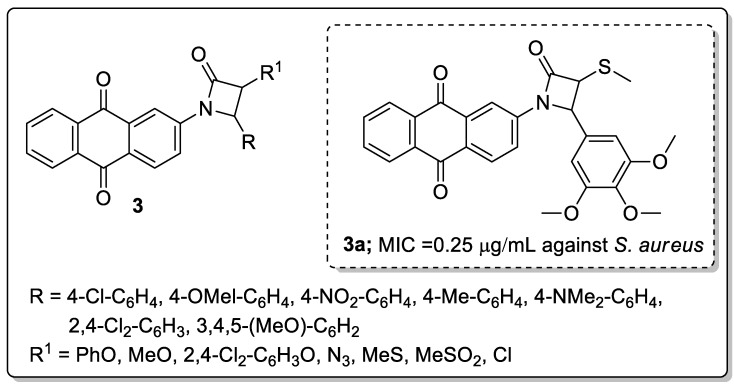
Most potent antibacterial β-lactam-anthraquinone hybrid **3a**.

A series of β-lactam-anthraquinone hybrids **3** were manufactured as potential antibacterial and antifungal agents [52]. Among them, the thio-methyl substituent at C-3 and the 3,4,5-trimethoxy phenyl group at the C-4 position of the β-lactam ring scaffold (compound **3a**) exhibited the most potent antibacterial activity (MIC = 0.25 μg/mL) against the *Staphylococcus aureus* bacterial strain, compared with the standard ciprofloxacin (MIC = 0.5 μg/mL) (Figure 5). The same compound displayed equal antifungal activity (MIC = 4 μg/mL) as the reference ciclopirox olamine (MIC = 4 μg/mL) against the *Candida albicans* strain. It was found that anthraquinone-β-lactam derivatives, which containing a 2-naphtho group at the C-3 position, and 4-chloro and 4-methoxyphenyl groups at C-4 of the β-lactam ring showed lower activity, which can be attributed to steric hindrance that weakens the intermolecular interactions. Furthermore, the active hybrid was evaluated by molecular docking studies and the carbonyl groups of the anthraquinone moiety and the β-lactam ring had three hydrogen bonding interactions with Lys273, Asp295 and Val277 residuces, while the MeS group interacted with the active site of Tyr272 and the 3,4,5-trimethoxyphenyl group exerted hydrophobic interactions with His293, Lys289, Gln292 and Lys319 residues of a penicillin-binding protein (PBP).

**Figure 6:**
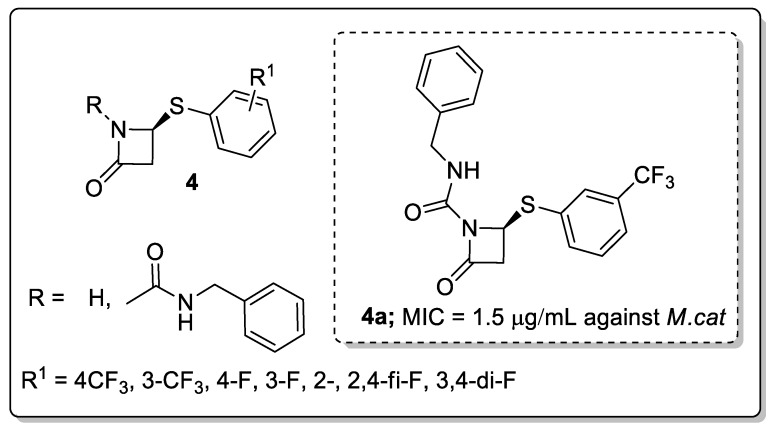
The antimicrobial activity of the most potent β-lactam analog **4a**.

β-Lactams and their derivatives **4** were synthesized and evaluated for antimicrobial activity against *Mycobacterium tuberculosis* (*M.tb*) and *Moraxella catarrhalis* (*M.cat*) [53]. Among them, the *meta*-CF_3_ of the phenylthiol ring and the achiral carbamyl group at the lactam nitrogen showed potent activity against *M.tb* (MIC = 25 μg/mL) and *M.cat* (MIC = 1.5 μg/mL) (Figure 6). In contrast, a decreasing activity was found with *para*-CF_3_, fluorine (*para-*, *meta-* and *ortho-*), and difluoro groups substituted on the phenylthiol ring analogs. Moreover, the addition of the achiral carbamyl group enhanced anti-Mtb activity relative to the unsubstituted derivative. The lack of a substantial difference in the activity against *M.cat* and *M.tb* may be due to nonspecific binding of the respective compounds to hydrophobic medium components. The active compound showed good activity against non-replicating and multi-drug resistant *Mtb*.

## 3. Five-Membered Ring Heterocycles

The five-membered heterocyclic motifs are known as 1,2,3-triazoles, imidazoles, pyrazoles, oxadiazoles, oxazoles, isoxazoles and thiazoles. Which are important pharmocophores in medicinal chemistry due to its exhbits broad spectrum of biological activities.

### 3.1. 1,2,3-Triazoles

**Figure 7:**
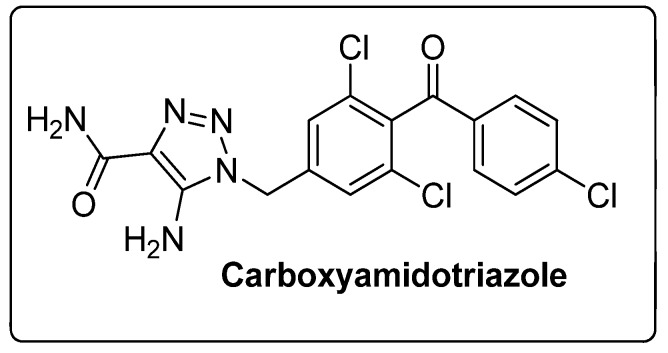
A 1,2,3-triazole-containing clinical drug.

The 1,2,3-triazole moiety is the main pharmacophore system among the nitrogen-based molecules and is a privileged building block in the discovery of various new biological targets. These five-membered heterocyclic motifs with three nitrogen heteroatoms can be easily prepared via ‘click’ chemistry, i.e., by copper-catalyzed azide-alkyne cycloaddition [Cu-AAC] reactions. Generally, the ‘linker’ property of 1,2,3-triazoles is stable to hydrolysis under acidic or basic conditions, and metabolic degradation. These compounds can interact with different biological targets through hydrogen bonding, noncovalent and van der Waals interactions as well as dipole-dipole bonding interactions. Furthermore, triazoles are weakly acidic and weakly basic and are more sensitive to reducing agents. Moreover, the 1,2,3-triazole-based compound, carboxyamidotriazole, has been successfully clinically evaluated for cancer treatment (Figure 7) [54,55,56]. Furthermore, the strong dipole properties of the triazole unit increased its importance in the field of medicinal chemistry, as it binds to the biological target with high affinity.

**Figure 8:**
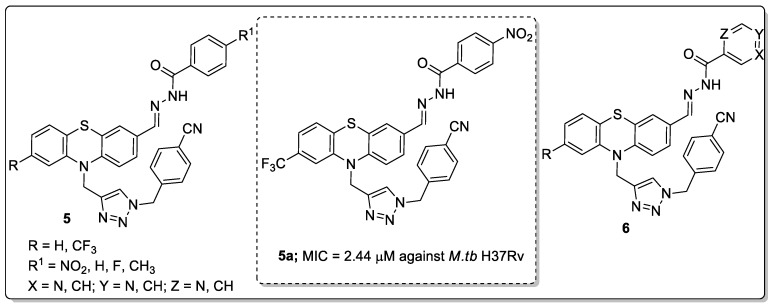
Most significant antitubercular activity phenothiazine-1,2,3-triazole conjugate **5a**.

Phenothiazine conjugates **5** and **6** with a 1,2,3-triazole linker were developed and the conjugates screened for anti-tubercular activity against *Mycobacterium tuberculosis* H37Rv strain [57]. All the compounds exhibited relatively good in vitro anti-tubercular activity as demonstrated by the compound **5a** bearing a 4-nitro group that showed significant anti-tubercular activity with a MIC value of 2.44 μM against *M.tb* H37Rv (Figure 8). Compound **5a** also displayed non-toxic characteristics against VERO cell lines. The isonicotinohydrazide/nicotinohydrazide derivatives **6** exhibited moderate activity with MIC values ranging from 2.61–2.94 μM against *M.tb* H37Rv. A SAR study revealed that the substituted phenyl rings, instead of the isonicotinohydrazide/ nicotinohydrazide resulted in highly potent activity, which was dependent on the substituents’ electronic effect on the phenyl ring. The active compound was comfortably docked into the Inh A and Cyp121 enzymes. Compound **5a** was exhibited distinct hydrogen bonding formation and pi-pi interactions with the hydrophobic residue Tyr158 of the Inh A enzyme. The most potent compound displayed higher permeability and aqueous solubility values than the respective analogs. In addition to, the pharmacokinetic parameters suggested that the compound exhibits good oral bioavailability. The potent compound signified a novel hybrid for the development of potential anti-tubercular agents.

**Figure 9:**
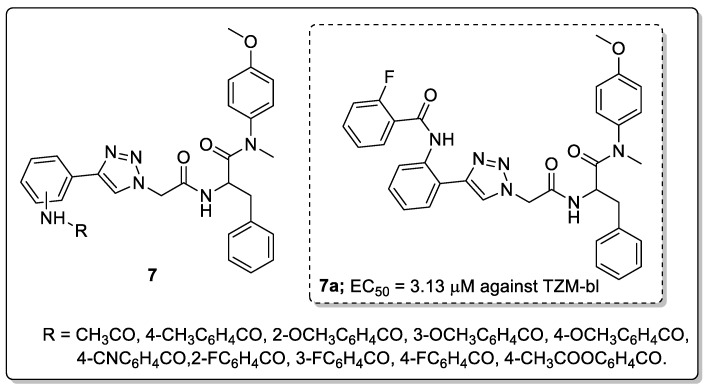
The anti-HIV activity of the most potent phenylalanine-1,2,3-triazole conjugate **7a**.

Phenylalanine scaffolds **7** with 1,2,3-triazole linkers were synthesized and tested for their antiviral activity in TZM-bl cells infected with the HIV-1 NL4-3 virus [58]. Most of the compounds displayed excellent activity with EC_50_ values ranging from 3.13–16.48 μM against HIV-1 in TZM-bl cells. Among them, the 2-fluoro benzamide compound **7a** exhibited high anti-HIV activity (EC_50_ = 3.13 μM) and showed very low toxicity (CC_50_ ≥ 16.48 μM, Figure 9). 

SAR studies revealed that the *ortho*-substituted aniline derivatives displayed higher activity than the *para*- and *meta*-substituted analogs. Particularly, fluorine substituted on the phenyl ring conjugates showed promissing antiviral activity. Furthermore, the binding mode of compound **7a** within the active site of the HIV-1 capsid (CA) monomer protein was confirmed. The triazole linked phenyl ring was formed a hydrophobic interaction with Met66 and an aliphatic hydrogen bonding could be formed between the methoxy group and Lys70 backbone in the first cluster and the Asn74 backbone in the second cluster. These results show that the large and smaller flexible groups of the 1,2,3-triazole moiety play a significant part in increasing the anti-HIV activity.

**Figure 10:**
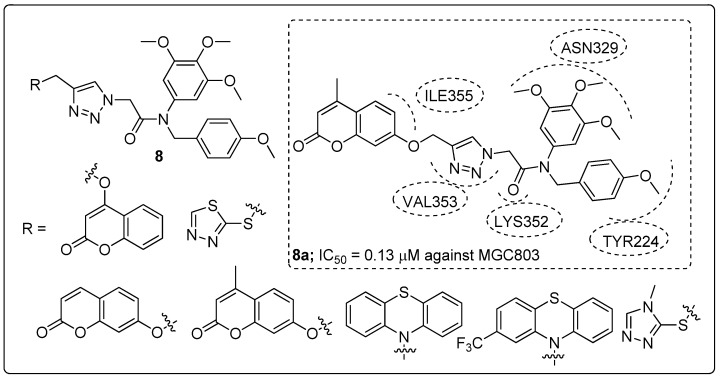
Antiproliferative activity of the most active 1,2,3-triazole scaffold **8a**.

1,2,3-Triazole-linked trimethoxyphenyl scaffolds **8** were prepared and screened for antiproliferative activity against prostate (PC3), liver (HepG2) and gastric (MGC803) cancer cell lines [59]. Among the synthesized hybrids, the coumarin moiety allied to a 1,2,3-triazole (compound **8a**) exhibited promising antiproliferative activity (IC_50_ = 0.13 μM) against the MGC803 cell line (Figure 10), compared with the standard drug colchicine (IC_50_ = 0.27 μM). Replacement of the coumarin moiety with a phenothiazine ring and were observed decreased activity. Compound **8a** could inhibit MGC803 cell growth and colony formation. It caused cell cycle arrest at the G2/M phase and tubulin polymerization was sturdily inhibited by correlating with the colchicine site. Furthermore, when the hybrid **8a** was validated by molecular docking studies it was found that the 3,4,5-trimethoxyphenyl ring, 4-methoxyphenyl ring, and the amide group formed three hydrogen bonding with the Asn329, Tyr224 and Lys352 residues at the active site of tubulin. These results revealed that coumarin with 1,2,3-triazole hybrids are potent colchicine site tubulin polymerization inhibitors.

**Figure 11:**
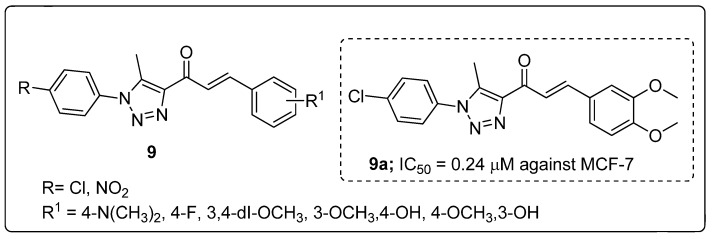
Most active anticancer agent chalcone conjugate with 1,2,3-triazole **9a**.

A library of chalcone conjugates **9** with a 1,2,3-triazole linker were synthesized and the conjugates screened for in vitro antiproliferative activity [60]. All the hybrids exhibited promising proliferative activity against a panel of 60 human cancer cell lines (NCI). The 3,4-dimethoxy and 4-chlorophenyl ring derivative **9a** displayed greater cytotoxic activity with IC_50_ values of 0.24 and 0.26 μM against the breast MCF7 and colon HCT116 cancer cell lines, respectively (Figure 11). The strong electron-withdrawing group (NO_2_) showed less activity than the chloro substituents. This observation may suggested that the electronic and steric properties of the substituents play an important role in the binding affinity of chalcones to their cellular target. The potent hybrid induced apoptosis via aggregation of MM RPMI-8226 cells in a dose-dependent manner and cell cycle arrest at the G2/M phase. Compound **9a** could be a potential lead for further advancement of anticancer studies and may offer new insights in treating multiple myeloma RPMI-8226.

**Figure 12:**
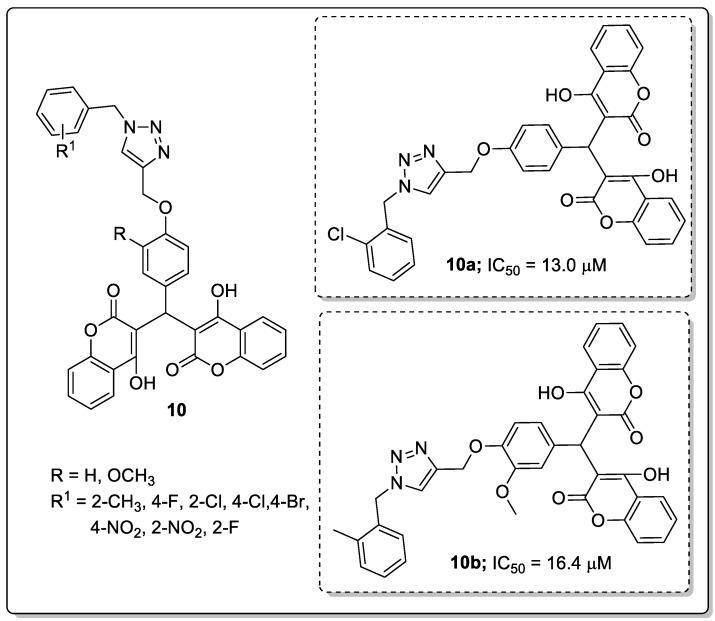
Most active 1,2,3-triazole-coumarin hybrids **10a** and **10b** as *α*-glucosidase inhibitors.

Biscoumarin compounds **10** with a 1,2,3-triazole linker were synthesized and screened the compounds for their in vitro *α*-glucosidase inhibitory activity as potential anti-diabetic agents [61]. Among them the screened hybrids, the compounds bearing with a 2-chloro (**10a**; IC_50_ = 13.0 μM) and 2-methyl (**10b**; IC_50_ = 16.4 μM) exhibited better *α*-glucosidase inhibitory activity than the standard drug acarbose (IC_50_ = 750.0 μM) (Figure 12). Changing the position of the chlorine atom in the phenyl ring from C-2 to C-4, led to a reduction in inhibitory activity. SAR studies revealed that the *ortho*-substituted phenyl hybrids showed highest activity than the *para*-substituted derivatives. The SAR evaluation also indicated electron-donating groups provided greater activity than electron-withdrawing groups. Furthermore, the compound **10a** was evaluated by molecular docking studies with active sites of the *α*-glucosidase enzyme. The results showed that the active compound **10a** formed a hydrogen bonding with residue Thr307, one of the coumarin rings of this compound formed a π-π interaction with His279 and the other coumarin ring interacted with Val305 via hydrophobic interaction, while the 2-chlorophenyl group established a π-anion interaction with Asp408 through phenyl ring and two hydrophobic interactions with Tyr313 and Phe311 through the 2-chloro substituent. These compounds are potential candidates for the development of novel anti-diabetic agents.

**Figure 13:**
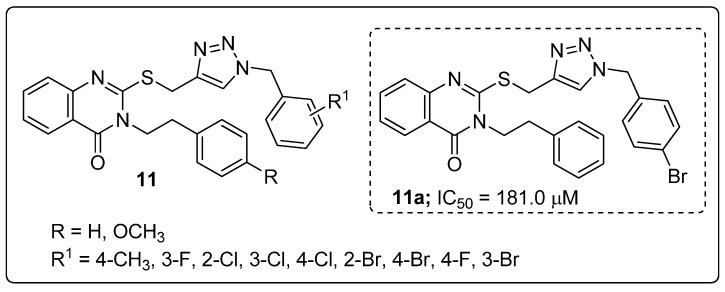
*α*-Glucosidase inhibitor activity of the most active 1,2,3-triazole-quinazolinone hybrid **11a**.

A series of quinazolinone hybrids **11** with a 1,2,3-triazole linker were reported by Saeedi and co-workers and the hybrids evaluated for their in vitro *α*-glucosidase inhibitory activity [62]. All the screened hybrids exhibited good inhibitory activity with IC_50_ values ranging from 181.0–474.5 μM compared with the reference acarbose with an IC_50_ value of 750.0 μM (Figure 13). Compound **11a** substituted with 4-bromobenzyl moiety exhibited highest *α*-glucosidase inhibitory activity (IC_50_ = 181.0 μM). SAR studies revealed that weak inhibitory activity was found when fluorine and chlorine atoms substituted the benzyl moieties unlike with the bromo compound **11a**. A slightly reduced activity was observed when the bromine atom was substituted on the *ortho* and *meta-*positions of the benzyl ring. Furthermore, by molecular docking studies in the active site of *α*-glucosidase, the quinazolinone moiety of hybrid **11a** was shown to form a hydrogen bonding and a π–π interaction with His279, and the phenethyl group and sulfur atom interacted with Arg312 residues. Hydrophobic interactions between Pro309 and the 1,2,3-triazole ring were also observed. Moreover, the 4-bromobenzyl group showed two interactions with Val305 and Val316 through the 4-bromo substituent and a hydrophobic interaction with Pro309 through the phenyl ring. Quinazolinone linked 1,2,3-triazole hybrids are potential conjugates for the treatment of antidiabetic activity.

**Figure 14:**
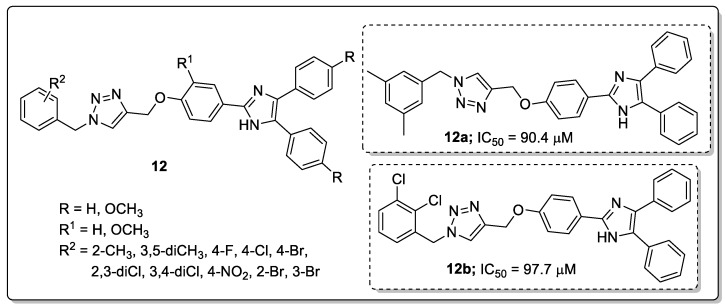
Most active 1,2,3-triazole-imidazole hybrids **12a** and **12b** as *α*-glucosidase inhibitors.

Saeedi’s group [63] investigated imidazole hybrids **12** with a 1,2,3-triazole linker and evaluated them for in vitro α-glucosidase inhibitory activity as a potential anti-diabetic agents. Among them, the phenyl ring hybrids with 3,5-dimethyl (**12a**) and 2,3-dichloro (**12b**) substitution showed the most potent inhibitory activity with IC_50_ values of 90.4 μM and 97.7 μM, respectively (Figure 14). 

Compounds **12a** and **12b** were 8-fold more effective than the reference acarbose (IC_50_ = 750.0 μM). Compounds bearing electron-donating groups showed higher inhibitory activity than those with electron-withdrawing groups. It can be concluded that the substituents on the benzyl moiety connected to the 1,2,3-triazole ring played a remarkable role for in vitro α-glucosidase inhibitory activity. Furthermore, the binding mode of the active hybrid **12a** fitted well into the active site of *α*-glucosidase with two hydrogen-bonding interactions between the NH of the imidazole moiety and Thr307, as well as the nitrogen of 1,2,3-triazole ring and Arg312. Furthermore, two hydrophobic interactions were notable between the 3,5-dimethylbenzyl moiety and the Phe300 and Arg439 residues.

**Figure 15:**
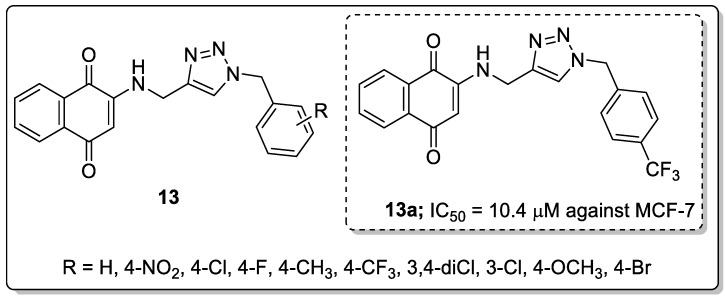
Cytotoxic activity of the most-active naphthoquinone-1,2,3-triazole conjugate **13a**.

Aminonaphthoquinone conjugates **13** bearing a 1,2,3-triazole linker were synthesized and the conjugates evaluated for their cytotoxicity activity against three human cancer cell lines, namely MCF-7, MOLT-4, and HT-29 [64]. The hybrid with a 4-trifluoromethyl group substituent on the benzyl moiety (compound **13a**) displayed excellent activity with an IC_50_ value of 10.4 μM against the breast cancer (MCF-7) cell line, as compared to cisplatin (IC_50_ = 8.8 μM) as a reference drug (Figure 15). The introduction of the 4-nitro group diminished the lipophilic character of the hybrid, making the compound completely inactive against all the three cancer cell lines. The active hybrid **13a** caused cell cycle arrest at the G0/G1 phase in the breast cancer MCF-7 cells. 

### 3.2. Imidazoles and Benzoimidazoles

**Figure 16:**
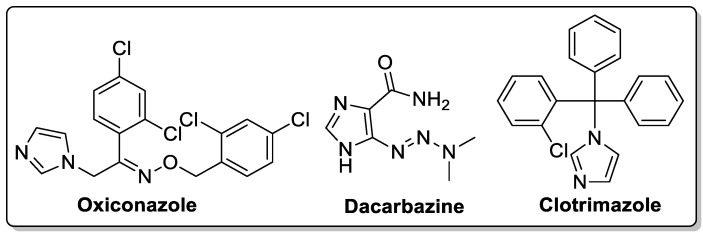
Imidazole clinical drugs.

The unique structural features of the five-membered imidazole and benzoimidazole moieties distinguish them as important heterocycles and these structures are part of many natural products and synthetic compounds. The electron-rich nature of the imidazole-based derivatives is useful to freely bind with various receptors and enzymes in the biological profile, thus showing broad biological activities [65,66]. Several imidazole-based molecules (oxiconazole, dacarbazine, and clotrimazole) exhibiting antifungal and anticancer activities with high therapeutic potency are clinically used as drugs (Figure 16). The scope of imidazole- and benzoimidazole-based molecules increasing rapidly in medicinal chemistry.

**Figure 17:**
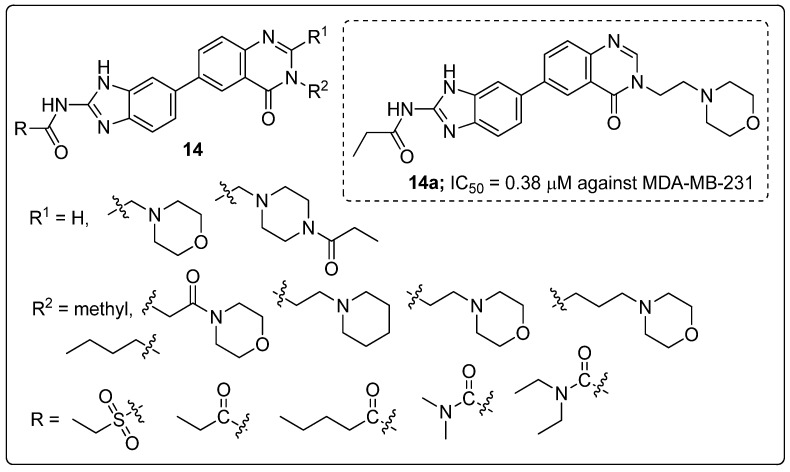
Cytotoxic activity of the most active benzoimidazole-quinazolinone hybrid **14a**.

Benzoimidazole-quinazolinone hybrids **14** were synthesized and tested the molecules for their cytotoxicity and Aurora-A kinase inhibitory activity by MTT assay [67]. All the tested hybrids showed good cytotoxicity activity, with IC_50_ values ranging from 0.38 to 18.13 μM against three cancer cell lines namely breast cancer (MDA-MB-231), prostate cancer (PC3) and neuroblastoma (SH-SY5Y) (Figure 17). Among them, the morpholinoethyl compound **14a** displayed excellent activity with an IC_50_ value of 0.38 μM (MDA-MB-231), 1.09 μM (PC3) and 0.77 μM (SH-SY5Y). Compound **14a** also showed promising inhibitory activity (IC_50_ = 21.94 μM) against Aurora-A kinase. In addition to, compound **14a** displayed induced G2/M phase cell cycle arrest and cell apoptosis via Aurora-A kinase inhibition. The replacement of the morpholinoethyl moiety with a methyl group might decrease the anticancer activity. Moreover, an extended propenamide alkyl side-chain at position 2 of the benzimidazole or an increased steric hindrance due to the introduction of a macrocyclic substituent may diminish the anticancer activity. Also, the replacement of propenamide with an electron-withdrawing group ethyl sulfonamide significantly reduced the anticancer activity. Furthermore, molecular docking studies revealed that the binding mode of compound **14a** formed hydrogen bonding interactions with the amino acid residues Ala213 in the kinase hinge region, the main chain NH of Thr217 and the catalytic lysine of Lys162. The quinazolinone fragment formed hydrophobic interactions including π-π stacked and π-alkyl interactions with different residues including Leu139, Ala160, Leu194, Leu210, Ala213 and Leu263 of Aurora-A. The benzoimidazole-quinazoline hybrids are promising candidates for the progress of potential anticancer agents.

**Figure 18:**
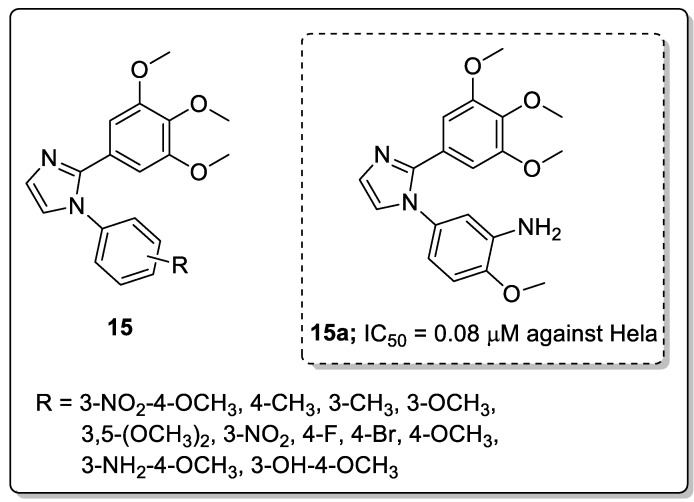
Antiproliferative activity of the most active 2-arylimidazole derivative **15a**.

Molecules **15** having 1-substituted-2-arylimidazoles were synthesized and screened for their antiproliferative activity against six cancer cell lines, namely breast (MDA-MB-468, MDA-MB-231 and T47D), colorectal (HCT15 and HT-29), cervical (Hela) and endothelial (HUVEc) and for tubulin polymerization inhibition [68]. Among the tested derivatives, the one with a 3-NH_2_ and 4-OCH_3_ substituted phenyl ring (compound **15a**) exhibited superior anticancer activity with IC_50_ values of 0.09 μM against the MDA-MB-468 cell line and 0.08 μM against the Hela cell line (Figure 18). The active compound caused G2/M phase cell arrest and cell apoptosis and strongly inhibited tubulin polymerization. Furthermore, in vivo studies of compound **15a** also demonstrated highly effective tumor growth inhibition of 77% at 60 mg/kg in an MDA-MB-468 cell. Introducing an electron-withdrawing group on the aromatic C-ring leads to a decrease in activity while an electron-donating group enhances the activity. Molecular docking studies of this compound **15a** indicated that it bound well in the colchicine binding site of tubulin and a hydrogen bonding was observed between the amino group of **15a** and the Thr179 of tubulin. These hydrogen bonds, together with the hydrophobic interactions provided by the aromatic rings, contribute to the high binding affinity of **15a** to tubulin, resulting in high activity. The diarylimidazole derivatives are promising candidates for the development of potential anticancer agents.

**Figure 19:**
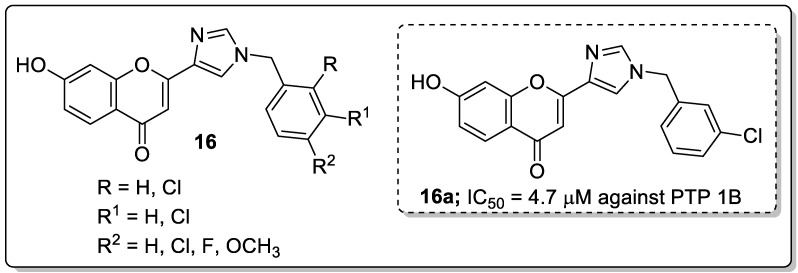
Most active imidazole flavonoid conjugate **16a** as a PTP1B inhibitor.

Imidazole and its flavonoid analogs **16** were synthesized and the analogs evaluated for their protein tyrosine phosphatase1B (PTP1B) inhibitory activity as potential antidiabetic agents [69]. All the hybrids showed good PTP1B inhibitory activity with IC_50_ values ranging from 0.63–5.0 μM, compared to oleanolic acid (IC_50_ = 4.7 μM). Among the series, compound **16a** bearing a 3-chloro group on the phenyl ring moiety exhibited potent PTP1B inhibitory activity with an IC_50_ value of 0.63 μM (Figure 19). Compound **16a** displayed a 9.5-fold high selectivity ratio for PTP1B over T-cell protein tyrosine phosphatase (TCPTP). Compound **16a** also presented low toxicity to a normal human embryonic kidney (HEK293) cell line. Reduced activity was observed with the replacement of the chlorobenzyl moiety by a fluorobenzyl group. The positional change of chlorine atom on the phenyl ring was not beneficial for the bioactivity either. The OH group of compound **16a** facilitated hydrogen bonding interactions with the Lys197 and Glu200 residues and also other hydrogen bonding interactions that formed through the nitrogen atoms of the imidazole ring with the Asn193 residue of PTP1B. The outcomes revealed that imidazole flavonoid scaffolds are potential inhibitors of PTP1B for the progress of antidiabetic agents.

**Figure 20:**
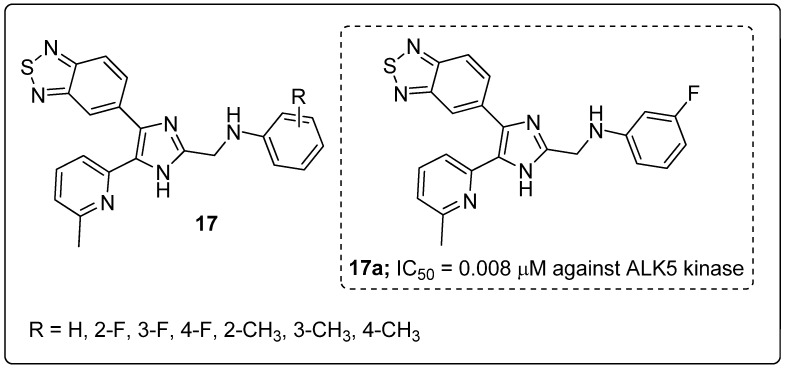
ALK5 inhibitory activity of the most active benzthiadiazole-imidazole scaffold (**17a**).

Series of benzo[1,2,5]thiadiazole-imidazole derivatives **17** were prepared and tested for their activin receptor-like kinase 5 (ALK5) inhibitory activity [70]. Among the series of compounds, the derivative **17a** substituted with a *meta*-fluoro group on the phenyl ring exhibited excellent inhibitiory activity (IC_50_ = 0.008 μM) against ALK5 kinase (Figure 20). SAR studies revealed that the strong electron-donating (CH_3_) groups of the derivatives showed less activity than the halogen-substituted on the phenyl ring derivatives. In addition, compound **17a** also strongly inhibited TGF-*β1*-induced Smad signaling in SPC-A1 and HepG2 cells. It showed however very low aqueous solubility, which limits oral administration, and its structure contains pyridine and imidazole moieties that can form salts in stomach acids, which can improve the water solubility. Based on the ADMET analysis the active compound **17a** showed good pharmacokinetic properties.

**Figure 21:**
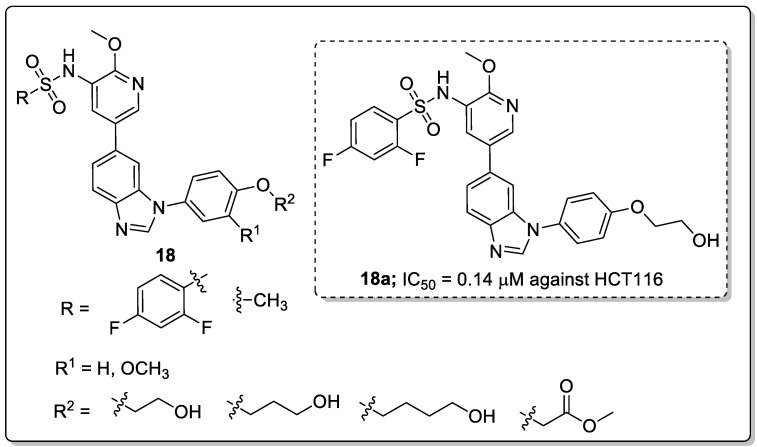
Antiproliferative activity of the most active benzo[*d*]imidazole conjugate **18a**.

A library of 1,6-disubstituted-1*H*-benzo[*d*]imidazole derivatives **18** was synthesized and the derivatives tested for their in vitro antiproliferative activity against the T47D, HCT116 and MCF-7 cancer cell lines [71]. Among them, the compound **18a** with a 2,4-difluoro substitution pattern on the sulfonyl phenyl ring showed significant activity, with IC_50_ values of 0.36, 0.14 and 0.31 μM against the T47D, HCT116 and MCF-7 cancer cell lines, respectively (Figure 21). 

SAR studies revealed that the difluoro-substituted derivatives have superior antiproliferative activity than the methyl-substituted derivatives. Introducing a methoxy group led to a decrease in the activity. In addition, the active compound **18a** effectively inhibited cell proliferation through suppression of PI3K kinase and blocking the PI3K/Akt pathway in HCT116 cells. Additionally, **18a** could inhibit the migration and invasion ability of HCT116 cells and could induce apoptosis of HCT116 cells. Molecular docking studies of this molecule **18a** could fit into the binding site of PI3K kinase. The nitrogen of the benzo[*d*]imidazole group formed a hydrogen bonding with the side-chain of Val882 in the hinge binder region of PI3K. Furthermore, the oxygen of the methoxy group formed an additional hydrogen bonding interaction with Lys833. Moreover, the nitrogen of the pyridyl group formed a hydrogen bonding with the conserved water molecule. In addition to these, the hydrogen of the hydroxyl group formed a hydrogen bonding interaction with Thr887. As evident from the ADMET investigation, compound **18a** displayed good drug-like properties. Thus, the benzoimidazole derivatives are promising candidates as potential PI3K inhibitors for anticancer drug advances.

**Figure 22:**
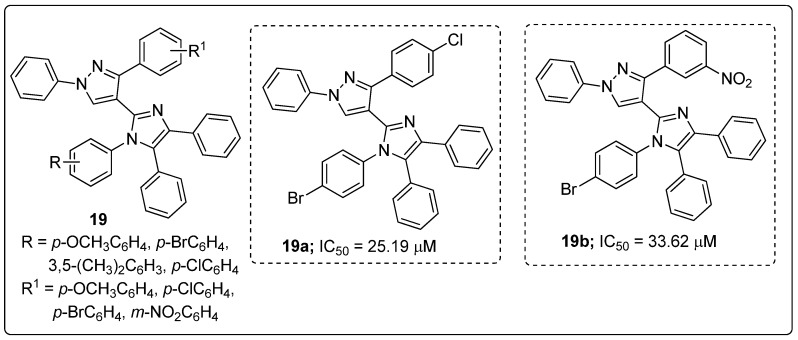
Most active pyrazole-imidazole hybrids **19a** and **19b** as α-glucosidase inhibitors.

Imidazole tethered pyrazole hybrids **19** were synthesized and the hybrids screened for their in vitro *α*-glucosidase enzyme inhibitory activity as potential antidiabetic agents [72]. The two hybrids with a bromine atom at the *para*-position of the phenyl ring attached to the imidazole nitrogen (compounds **19a** and **19b**) displayed the highest inhibitory activity with IC_50_ values of 25.19 μM and 33.62 μM against the α-glucosidase enzyme, respectively, as compared with the standard acarbose (IC_50_ = 38.25 μM, Figure 22). SAR studies revealed that the halogen (Br and Cl)-substituted hybrids have superior inhibitory activity than those with electron-donating groups substituents. Molecular docking studies of the molecules **19a** and **19b** showed that they interact with the binding pocket of the α-glucosidase enzyme, and a hydrogen bonding interaction was observed between the unsubstituted nitrogen atom of pyrazole ring and amino acid Asn24. Moreover, the oxygen atoms of the nitro group were acting as hydrogen bonding acceptors towards the amino acid Arg312.

### 3.3. Pyrazoles

**Figure 23:**
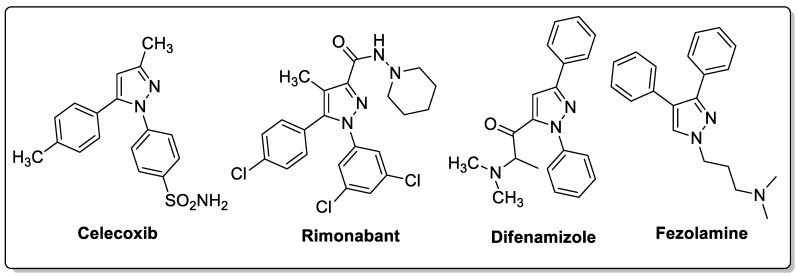
Pyrazole-based clinical drugs.

Pyrazole is a well-known five-membered nitrogen-based heterocycle and exhibits a broad spectrum of synthetic and biological applications. Several pyrazole-based drugs namely celecoxib, rimonabant, difenamizole and fezolamine, etc., with excellent anti-inflammatory, anti-obesity, analgesic and/or antidepressant activities have been developed and are used to treat various diseases (Figure 23) [32,33,34,35].

**Figure 24:**
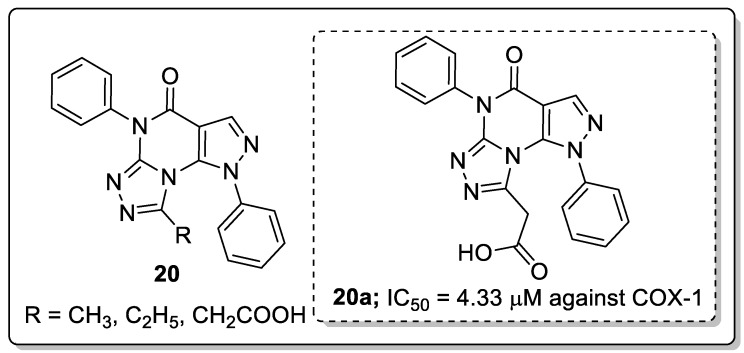
Anti-inflammatory activity of the most active pyrazole fused triazole hybrid **20a**.

Pyrazole fused triazole molecules **20** were identified as anti-inflammatory agents and COX-1/COX-2 enzyme inhibitors by Tageldin and co-workers [73]. Among them, compound **20a** showed promising anti-inflammatory activity with an IC_50_ value of 4.33 μM against the COX-1 enzyme as compared with celecoxib (IC_50_ = 5.46 μM) (Figure 24). SAR studies revealed that the less activity was observed with alkyl substituent derivatives than the acetoxy derivatives. Furthermore, docking studies of the most active compound showed that the acetate moiety of **20a** was involved in a H-bonding interaction with the Tyr385 of the COX-2 enzyme. In addition, the 1-phenyl substituent attached to the core pyrazolo[4,3-*e*][1,2,4]triazolo[4,3-*a*]pyrimidin appeared to display a hydrophobic interaction with the hydrophobic side chain of Trp387, Met522 and Phe518 residues.

**Figure 25:**
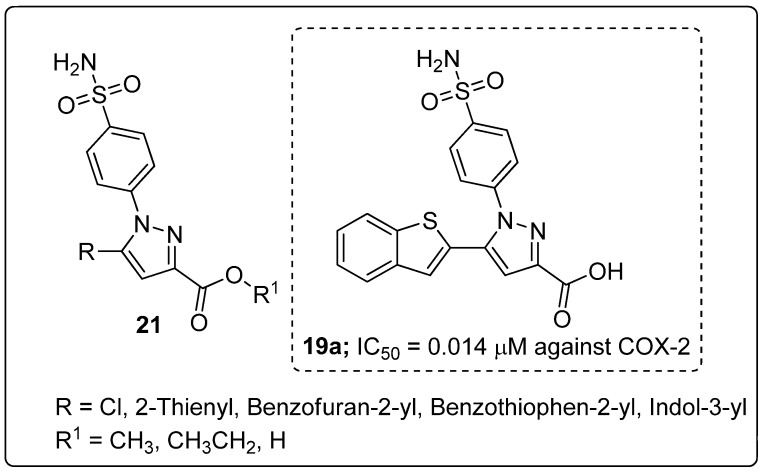
Anti-inflammatory activity of the most active pyrazole sulfonamide conjugate **21a**.

Pyrazole sulfonamide conjugates **21** were prepared and evaluated for their in vitro analgesic and dual COX-2/5-LOX inhibitory activity [74]. The pyrazole derivative with a benzothiophene (conmpound **21a**) exhibited potent inhibitory activity with the IC_50_ values of 5.40 μM (COX-1), 0.014 μM (COX-2) and 1.78 μM (5-LOX) (Figure 25). Compound **21a** showed equal or more potent analgesic activity (88.8% protection) than the standards indomethacin (84.4%) and celecoxib (31.1%), with no gastric ulcerogenic properties. 

Furthermore, the binding mode of the active compound **21a** was evaluated by molecular docking studies, where the negatively charged carboxylate moiety at the position 3 of the pyrazole compound was shown to be anchored in the active site through ionic interaction with the positively charged non-heme iron. Through hydrophobic interaction, the aryl substituent at position 5 interacts with the hydrophobic side-chains of the amino acids Leu368, Leu414, Ile415 and Phe421. In addition, the aryl substituent and the benzenesulfonamide moieties are involved in Van der Waals interactions with the amino acid Phe421. Through hydrogen bonding, the sulfonamide moiety interacts with Tyr181 and Asn425 residues in 5-LOX enzyme.

**Figure 26:**
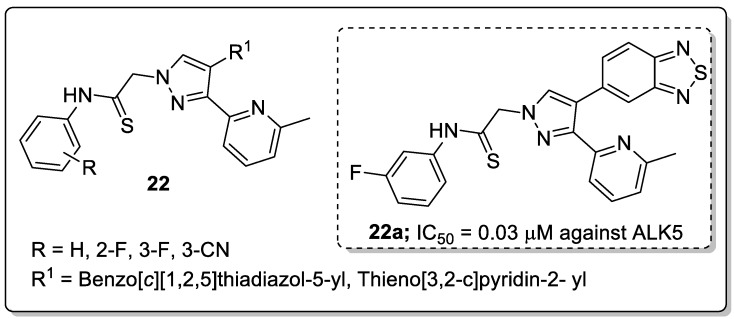
ALK5 kinase inhibitory activity of the most active pyridine-pyrazole derivative **22a**.

A library of pyridine-pyrazole derivatives **22** was synthesized and the new molecules screened for their activin receptor-like kinase 5 (ALK5) and p38a mitogen-activated protein (MAP) kinase inhibitory activities [75]. Among the screened compounds, the benzo[*c*][1,2,5]thiadiazol-5-yl linked pyrazole along with the *meta*-fluoro phenyl derivative **22a** exhibited high inhibitory activity (IC_50_ = 0.030 μM) against ALK5 kinase, which was 4-fold greater activity than that of the clinical drug LY-2157299 (IC_50_ = 0.119 μM, Figure 26). Replacement of benzo[*c*][1,2,5]thiadiazol-5-yl with a thieno[3,2-*c*]pyridin-2-yl moiety resulted in reduced activity. In addition, pyrazoles possessing the benzo[*c*][1,2,5]thiadiazol-5-yl moiety, with the introduction of fluoro- or carbonitrile substituents at the *ortho*- or *meta*-position of the phenyl ring showed decreased ALK5 inhibitory activity. Western blotting and RT-PCR assays revealed that compound **22a** strongly inhibited TGF-*β* induced LX-2 human hepatic stellate cell HSC activation and efficiently suppressed mRNA expressions of collagen I and *α*-SMA. The active compound **22a** fitted well into the binding site of ALK5 kinase. The two rings of the benzo[*c*][1,2,5]thiadiazol-5-yl moiety formed a π-alkyl bonding with Leu340. Lys232 formed a π-alkyl bonding with the pyridine ring in **22a** and the benzene ring of Tyr249 formed a π-alkyl bonding with the methyl group of the pyridine ring. The NH group of the side-chain in **22a** formed a hydrogen bonding with the carbonyl group of the Lys337 and Asn338, and with the carboxylic acid of the side chain in Asp351. The phenyl ring of the side-chain formed a π-alkyl bonding with the Lys337 residue.

**Figure 27:**
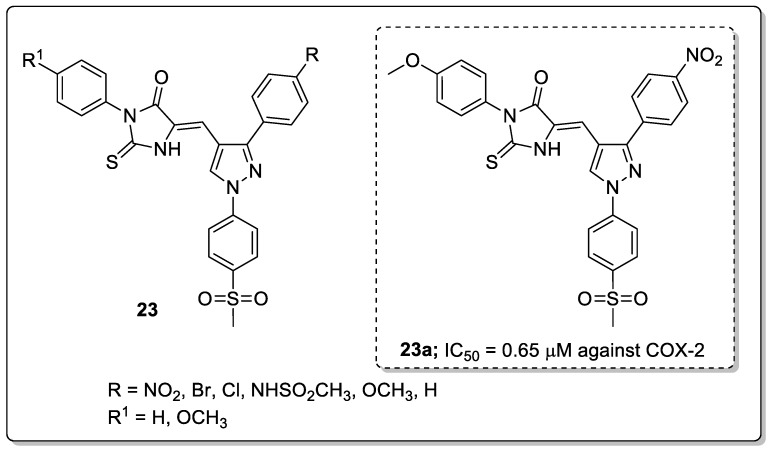
Anti-inflammatory activity of the most active pyrazole-thiohydantoin conjugate **23a**.

Pyrazole-linked thiohydantoin derivatives **23** were synthesized and tested for their anti-cancer and anti-inflammatory activities [76]. Among them, the nitro substituted compound **23a** showed high anti-inflammatory activity (IC_50_ = 0.65 μM) against the COX-2 enzyme as compared with celecoxib (IC_50_ = 0.84 μM) (Figure 27). The compound exhibited significantly less ulcerogenic activity (ulcer index = 3.21) than the non-ulcerogenic celecoxib as the reference drug (ulcer index = 2.99). In addition to that, a series of compounds were evaluated for their cytotoxic activity against lung carcinoma (A-549), colon (HCT-116) and breast (MCF-7) cancer cell lines. The same compound **23a** exhibited the most potent cytotoxic activity (IC_50_ = 3.73 μM) against the human colon (HCT-116) cancer cell line. Compound **23a** inhibited human topoisomerase-1 (Topo-1) with an IC_50_ value of 29.7 μg/mL, as compared with the reference camptothecin (IC_50_ = 20.2 μg/mL). The methoxy-substituted derivative has higher cytotoxic activity than the respective unsubstituted derivatives. Furthermore, the binding mode of the active compound **23a** with COX-2 and human topoisomerase-1 enzyme was investigated by docking studies. Compound **23a** interacts via hydrogen bonding with Tyr341, Tyr371, Ser516 residues of COX-2. Also, there is a hydrogen bonding interaction with the Asp533 and two π-π stacking interactions with DNA base pairs through arene-arene interactions with deoxyadenosine DA113 of the human topoisomerase-1 enzyme. It can be concluded that for these types of analogs, the presence of a *para*-substituted methoxy in addition to the COX-2 pharmacophore SO_2_CH_3_ is important for binding with both COX-2 and human Topo-1. The pyrazole-thiohydantoin derivatives are promising candidates for the development of anti-inflammatory and anti-cancer agents.

**Figure 28:**
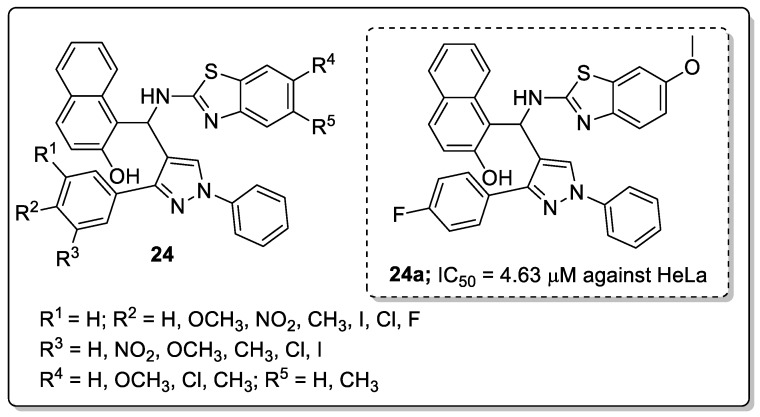
Antiproliferative activity of the most active pyrazole-benzothiazole-*β*-naphthol hybrid **24a**.

Pyrazole-tagged benzothiazole-*β*-naphthol hybrids **24** were prepared and evaluated for their in vitro antiproliferative activity against four (A549, MCF 7, HeLa and HEK-293) human cancer cell lines [77]. Amongst them, compound **24a** bearing a fluorine atom at the *para*-position on the phenyl ring moiety showed the most significant antiproliferative activity against the human epithelial cervical (HeLa) cancer cell line (IC_50_ = 4.63 μM) (Figure 28). SAR studies revealed that the compound with electron-withdrawing groups exhibited greater activity than those with electron-donating groups. Compound **24a** caused G2/M phase cell cycle arrest and effectively inhibited Topo-1 and was shown to strongly bind to DNA. Furthermore, docking studies showed that the compound significantly interacts with topoisomerase-I and DNA by binding to its minor groove.

**Figure 29:**
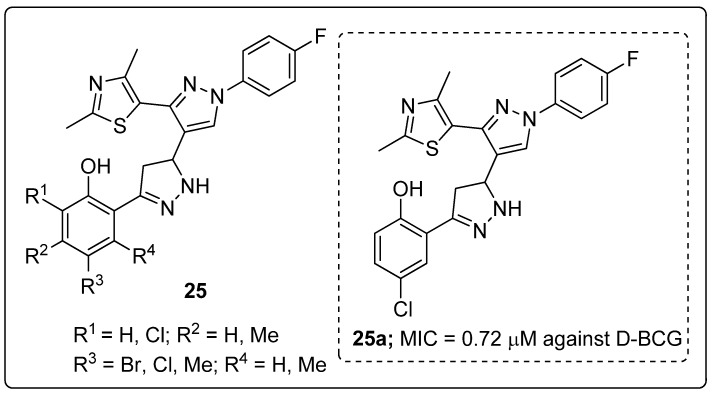
The antitubercular activity of the most active thiazole-pyrazole hybrid **25a**.

Pyrazole-linked thiazole hybrids **25** were synthesized and evaluated for their antimycobacterial activity against dormant *M. tuberculosis* H37Ra (D-MTB) and *M. bovis* BCG (D-BCG) [78]. The hybrid **25a** substituted with a *meta*-chloro group on the phenyl ring exhibited the most siginificant *M. tuberculosis* activity with MIC values of 1.16 μM against D-MTB and 0.72 μM against D-BCG (Figure 29). SAR studies revealed that the dichloro and bromo groups substituted on the phenyl ring derivatives were inactive. Furthermore, the active compound **25a** exhibited low cytotoxicity against the human HeLa, PANC-1 and A549 cancer cells. These hybrids are potential candidates for the progress of antitubercular agents.

**Figure 30:**
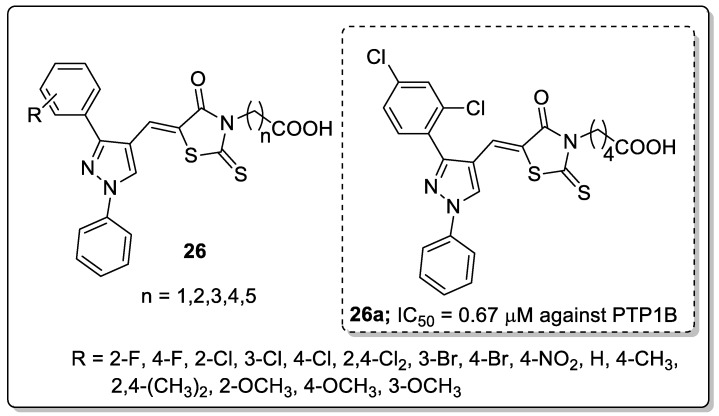
PTP1B inhibitory activity of most active pyrazole conjugate **26a**.

Pyrazole and its 1,3-diphenyl analogs **26** were synthesized and the new molecules tested for their protein tyrosine phosphatase1B (PTP1B) inhibitory activity [79]. All the tested compounds exhibited good inhibitory activity with IC_50_ values ranging from 0.67–24.56 μM (Figure 30). The 2,4-dichloro with butyl linker derivative **26a** exhibited superior inhibitory activity (IC_50_ = 0.67 μM) against the PTP1B enzyme as compared to the standard oleanolic acid (IC_50_ = 1.62 μM). The change in substitution on the phenyl ring and linker length influenced the activity. No activity was found for the fluoro- and methoxy-substituted on the phenyl ring molecules and a linker length of four. In addition, a 9-fold selectivity was observed against PTP1B over TCPTP enzymes. Furthermore, the active compound **26a** was evaluated by the molecular docking studies and was fitted well into the catalytic site of the PTP1B enzyme. The carbonyl and hydroxyl moieties of the carboxyl groups that are known as hydrogen bonding acceptors and donors, formed three hydrogen bonding with the backbone of Ser216 and the side-chains of Arg221 and Cys215 in PTP1B. Some van der Waals interactions, such as interactions between the terminal benzene ring and Gly259 and Arg24, the sulfur atom of the carbon-sulfur double bond of the thiazole ring and Met258, and the sulfur atom of the thiazole ring and Asp48 residues in PTP1B were also observed.

**Figure 31:**
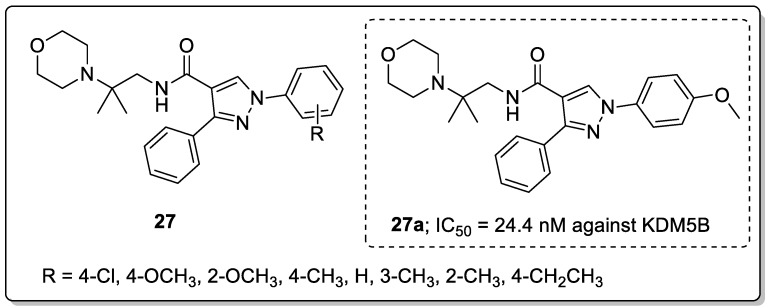
KDM5B inhibitory activity of the most active pyrazole conjugate **27a**.

Pyrazole derivatives **27** were synthesized and screened for their KDM5B inhibitory activity as potential gastric cancer agents [80]. Among the tested derivatives, the 4-methoxylbenzyl-substituted compound **27a** exhibited significantly increased potency against KDM5B with an IC_50_ of 24.4 nM (Figure 31). The presence of an electron-donating 2-methoxybenzyl group decreased the activity compared with the 4-methoxy derivative. The *para*-substituted derivatives showed greater potency than the *ortho*-substituted derivatives. Due to the *p*-methoxyphenyl group in the junction of the hydrophobic and hydrophilic pocket, the phenyl was surrounded by Tyr488, Tyr425, and Val489 residues. In addition, **27a** was a potent KDM5B inhibitor with acceptable selectivity against KDM5A/B/C, but it performed poorly against KDM4A/C and KDM6B. The capability of compound **27a** to stabilize KDM5B in a dose-dependent manner and induce the accumulation of H3K4me2/3 in the gastric cancer cell line MKN45, supports the use of compound **27a** as a potent and cellular active KDM5B inhibitor. Furthermore, the compound showed potent cellular active KDM5B inhibition and inhibited MKN45 cell proliferation, wound healing and migration. The molecular docking studies showed that molecule **27a** fitted well in the tight flat binding pocket of KDM5B. The oxygen atom of the methoxy group formed a hydrogen bonding with the Lys517, and the nitrogen of amide had a hydrogen interaction with Tyr488 and π-π stacking interactions with the benzene ring of Tyr488 residues of KDM5B. The pyrazole derivatives are promising candidates for targeting KDM5B inhibition and as a new therapeutic strategy for gastric cancer treatment.

**Figure 32:**
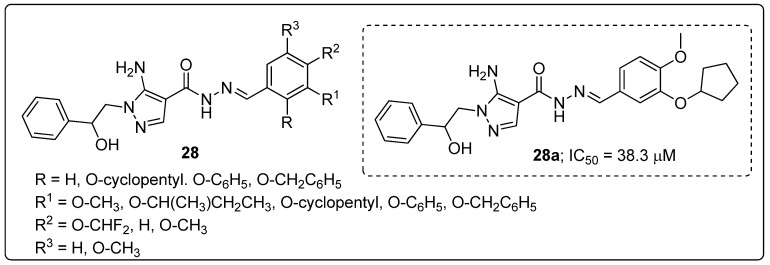
ROS inhibitory activity of the most active pyrazole conjugate **28a**.

Pyrazole scaffolds **28** were synthesized and the scaffolds evaluated for their reactive oxygen species (ROS) inhibitory activity on human platelets [81]. Most of the tested compounds were able to inhibit ROS production with IC_50_ values of 8.6 to 38.3 μM. Among the established molecules, the O-cyclopentyl and O-CH_3_ compound **28a** showed the most active ROS production inhibition with an IC_50_ value of 38.3 μM (Figure 32). 

Reduced activity was observed with a tri-methoxy substituted on the phenyl ring derivative than for all the tested compounds. In addition, compound **28a** strongly inhibited both PDE4D3 (IC_50_ = 1.05 μM) and PDE4B2 (IC_50_ = 0.55 μM) enzyme isomers. The results revealed that the pyrazole derivatives could block ROS production on human platelets, that produce high levels of ROS upon thrombin stimulation by a PDE4-independent mechanism.

## 4. Six-Membered Ring Heterocycles

### 4.1. Quinolines

**Figure 33:**
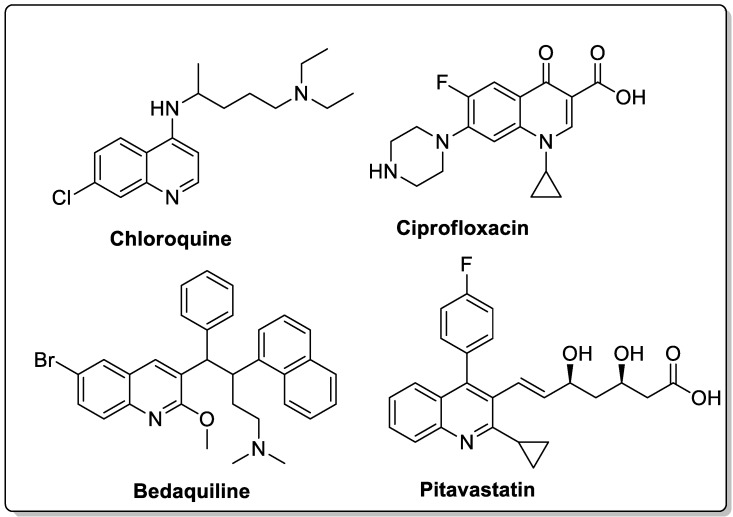
Quinoline clinical drugs.

The quinoline moiety is a well-known entity and is a ubiquitous alkaloid subunit of many natural products. Quinoline is an important pharmacophore moiety as it has been described to possess various biological activities, which include antimalarial, antibiotic, antitubercular, antiproliferative, antiprotozoal, antihypertensive and anti-HIV properties [82,83,84]. Currently various clinical drugs available in the market contain this moiety (Figure 33).

**Figure 34:**
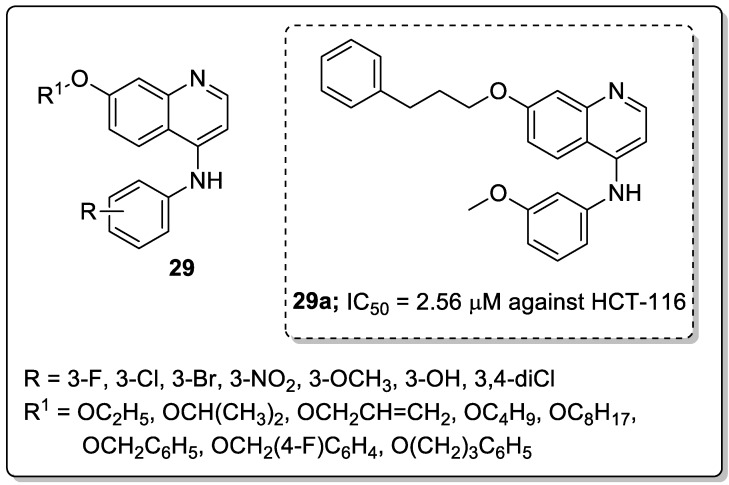
Antiproliferative activity of the most active quinoline conjugate **29a**.

Quinoline conjugates **29** were synthesized and evaluated for their antiproliferative activity [85]. Among them, the compound **29a** with a *meta*-methoxy on the phenyl ring and 3-phenylpropoxyl group exhibited the high antiproliferative activity against colorectal carcinoma (HCT-116) and cervical carcinoma (Hela) with IC_50_ values of 2.56 μM and 2.71 μM respectively (Figure 34). Compound **29a** was strongly inhibited tumor growth (82.1%) in mice bearing CT25-C126 cells and induced autophagy in colon cancer cells. It can be concluded that a large and bulky substituent in position-7 might be an advisable pharmacophoric group for antiproliferative activities. The novel quinoline analogs are potential candidates for the progress of antitumor agents.

**Figure 35:**
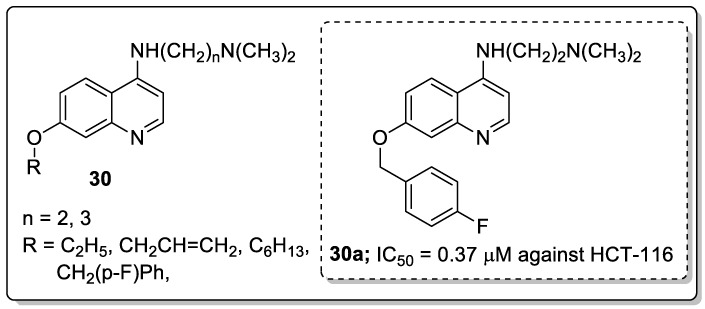
Antiproliferative activity of the most active quinoline conjugate **30a**.

Quinoline derivatives **30** were prepared and screened for antiproliferative activity [86]. The derivative **30a** with a 4-fluorobenzyloxy group and a flexible amino side-chain with a two methylene spacer in position-4 exhibited the most promising antiproliferative activity against all the tested cancer cell lines, namely, HCT-116, RKO, DLD1, HepG2, BGC-823, NCI-H1650 and SK-OV-3 with IC_50_ values of 0.37, 0.58, 0.81, 0.79, 0.89, 0.78 and 0.89 μM respectively (Figure 35). The active compound **30a** effectively induced p53-dependent cell apoptosis by targeting p53 transcriptional activity and reduced the viability of HCT116, DLD1 and RKO cells in a time-dependent manner in the CCK8 assay. In addition, compound **30a** significantly inhibited tumor growth in a colorectal cancer xenograft model in nude mice. The compounds bearing a flexible amino side-chain with a two-methylene spacer displayed more potent cytotoxic potency than compounds that contained a three-methylene spacer. The results indicated that the amino side-chain substituents are beneficial pharmacophoric groups for enhancing the antiproliferative activity. Thus, quinoline scaffolds are potential compounds for the progress of antitumor agents.

**Figure 36:**
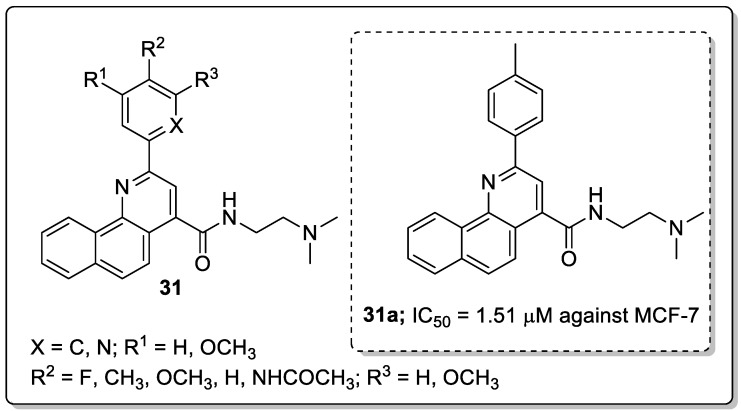
Antiproliferative activity of most active tetrahydrobenzo-quinoline scaffold **31a**.

Tetrahydrobenzo-quinoline scaffolds **31** were prepared and tested for their antiproliferation activity against the four human cancer cell lines MCF-7, C26, A2780 and A549 [87]. The compound bearning with a *p*-methyl group on the phenyl ring (**31a**) showed the most significant antiproliferative activity against the human breast cancer cell line (MCF-7) with an IC_50_ value of the 1.51 μM, as compared with the standard doxorubicin (IC_50_ = 0.5 μM) (Figure 36). The compounds possessing small lipophilic electron-donating substituents in the *para*-position of the phenyl ring showed higher cytotoxic activity than the other quinolines. The pyridine derivatives did not show any improved cytotoxic effect, which can be due to the electron-withdrawing character of the pyridine ring. Compound **31a** displayed significant DNA intercalating effects and effectively induced apoptosis in a dose-dependent manner. Furthermore, the authors undertook by molecular docking studies of the active compound **31a**, and the interaction of this compound with nucleobases involved hydrogen bonding between the NH groups of compound **31a** and adenine of the DNA backbone. The phenyl ring of compound **31a** showed hydrophobic and Vander Waals interactions with two thymine groups of DNA.

**Figure 37:**
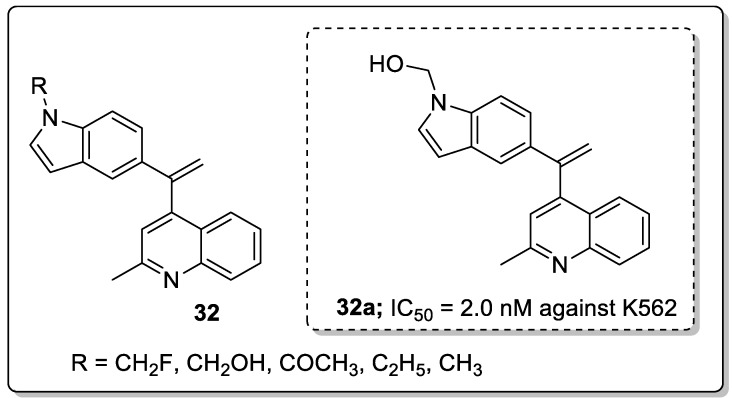
Antiproliferative activity of the most active indole-quinoline hybrid **32a**.

Quinoline-indole-hybrids **32** were synthesized and tested for antiproliferative activity [88]. Compound **32a** with a hydroxymethyl on the indole ring showed the most potent activity with an IC_50_ value of 2 nM against K562 cancer cells (Figure 37). Compound **32a** caused cell cycle arrest at the G2/M phase and induced apoptosis. In addition, **32a** effectively inhibited tumor growth in H22 xenograft models with no toxicity. Furthermore, compound **32a** binds well into the colchicine binding site of tubulin. The N-1 of the quinoline moiety formed hydrogen bonding with the residue of Cys241 and the indole rings extended into the hydrophobic pocket, which was surrounded by the residues Thr179, Val315, Asn350 and Val351. The hydroxymethyl group of **32a** formed two additional hydrogen bonds with Val315 and Asn350 residues. The novel quinoline hybrids are potential hybrids for the development of anti-tubulin agents.

**Figure 38:**
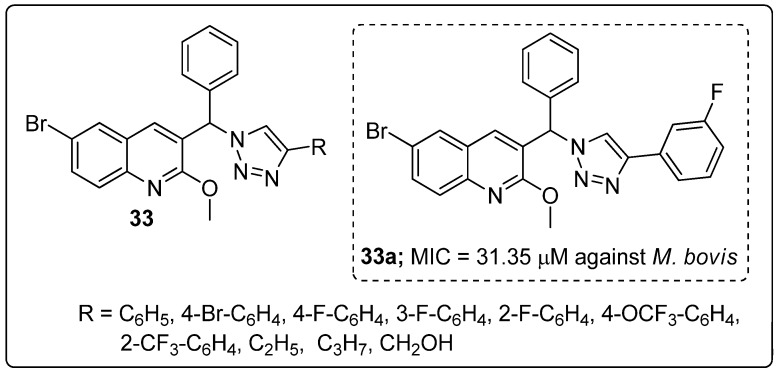
The antitubercular activity of the most active quinoline-triazole hybrid **33a**.

Quinoline hybrids **33** with a 1,2,3-triazole linker were synthesized and the new molecules screened for their antitubercular activity against *Mycobacterium bovis* [89]. Among the series of hybrids, compound **33a** bearing a 3-fluoro on the phenyl ring showed the most potent antitubercular activity with a MIC value of 31.35 μM, as compared to the standard isoniazid (MIC = 12.52 μM) (Figure 38). The presence of fluorine on the *meta*-position of the phenyl ring showed superior activity, whereas *ortho*- and *para*-fluoro phenyl did not display any activity. Furthermore, these hybrids tested for cytotoxicity and the active compound **33a** showed no growth inhibition for all the tested cell lines HeLa (cervical), PC3 (prostate), Panc-1 (pancreatic) and SKOV3 (ovarian).

**Figure 39:**
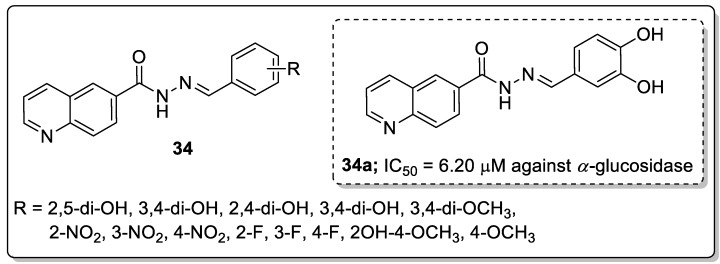
The most active quinoline with Schiff base analog **34a** as an *α*-glucosidase inhibitor.

Schiff bases of quinoline analogs **34** were synthesized and tested for their anti-diabetic activity against the *α*-glucosidase enzyme [90]. All the screened molecules exhibited good inhibitory activity with IC_50_ values ranging from 6.20–48.50 μM. Among them, the 3,4-dihydroxy conjugate **34a** displayed potent inhibitory activity (IC_50_ = 6.20 μM) against the *α*-glucosidase enzyme (Figure 39). The active compound **34a** showed a 6-fold greater potency than the reference acarbose (IC_50_ = 38.45 μM). SAR studies revealed that no inhibitory activity was noticed in the presence of electron-donating groups. The position of the hydroxyl moiety plays a role in the activity of these compounds, the relocation of OH groups from 3 and 4-position and their respective presence at the phenyl ring 2,5- and 2,3-positions decreased the inhibitory activity and showed lower inhibitory potential than analog **34a**. The hydroxyl moieties present in the *para*-position of the phenyl ring showed more interactions with the active site residues. The methoxy moiety instead of the hydroxyl moiety showed fewer interactions with active site residues as well as less activity. Furthermore, the docking studies of compound **34a** validated that the oxygen atom of the hydroxyl moiety of the phenyl ring of the compound formed a hydrogen bonding interaction with the active site residues of Lys126 and Glu171 of the *α*-glucosidase enzyme. The quinoline-Schiff-based derivative is a potential inhibitory agent for the treatment of anti-diabetic activity.

**Figure 40:**
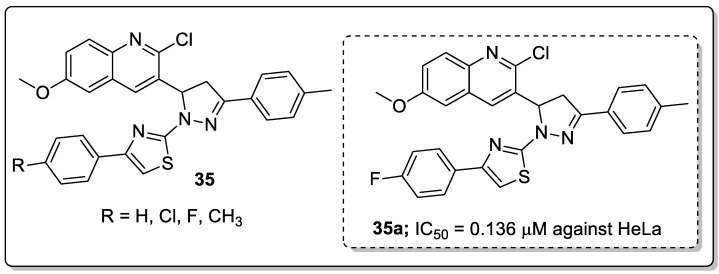
Antiproliferative activity of the most active quinoline-pyrazole-thiazole hybrid **35a**.

Hybrid molecules **35** having quinoline, pyrazole and thiazole moieties were synthesized and evaluated for their antiproliferative activity [91]. Among them, the hybrid **35a** with a *para*-fluoro substituent on the phenyl ring showed excellent antiproliferative activity (IC_50_ = 0.136 μM) against the cervical (HeLa) cancer cell line (Figure 40). The authors further reported epidermal growth factor receptor (EGFR) inhibitory activity and the active compound showed EGFR inhibitory activity (IC_50_ = 31.8 nM) at a nanomolar level as compared to the gefitinib standard drug (IC_50_ = 29.16 nM). Furthermore, compound **35a** displayed H-bonding with Met769, water-mediated H-bonding with Thr766 and a cation-π interaction with Lys721 residues. Compound **35a** also showed a hydrophobic interaction by the phenyl moiety with the hydrophobic side-chains of the amino acids Val702, Ala719, Met742 and Leu764 residues of EGFR active site. Therefore, the binding mode of the most active compound at the active site of EGFR confirmed its inhibitory activity, and the potential to act as an anti-proliferative agent through EGFR inhibition.

**Figure 41:**
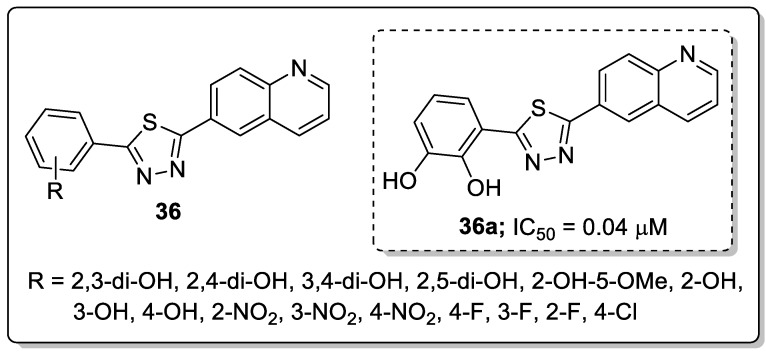
The antileishmanial potential of the most active quinoline-thiadiazole hybrid **36a**.

Another series of quinoline-linked to thiadiazole hybrids **36** were synthesized and the hybrids screened for their antileishmanial potential [92]. All twenty hybrids exhibited good antileishmanial potential with IC_50_ values ranging from 0.04–9.60 μM (Figure 41). Compound **36a** with a 2,3-dihydroxy group exhibited significant inhibition with an IC_50_ value of 0.04 μM, as compared with the pentamidine standard drug (IC_50_ = 7.02 μM). The superior potential shown by these compounds might be due to the hydroxyl that may be involved in hydrogen bonding. In contrast, halogen-substituted derivatives diminished the antileishmanial potential. Furthermore, compound **36a** fitted well in the active site of the pteridine reductase 1 (PTR1) enzyme. The two hydroxyl groups attached to the phenyl ring of the compound forming H-bonds with ArgA17 and AspA181, and the phenyl ring showed a π-interaction with the ArgA17 residue. The–NH of the ArgA17 interacts through its H with the nitrogen of the 1,3,4-thiadiazole moiety of the active compound **36a**. The strong bonding network of the compound with the residues of the active pocket might be one of the reasons for its excellent biological activity.

### 4.2. Quinazolines

**Figure 42:**
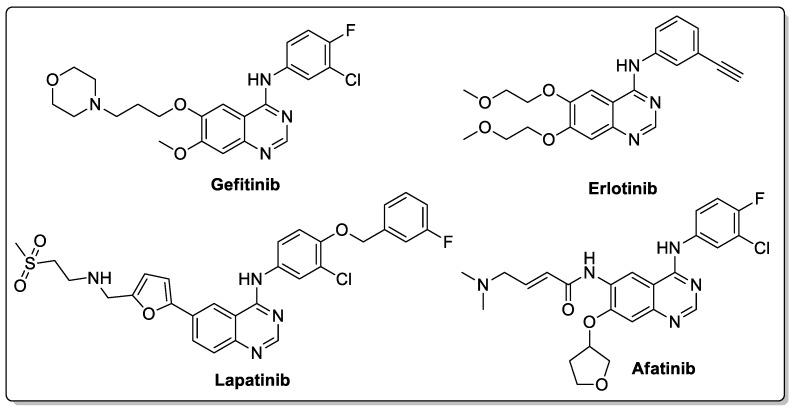
Quinazoline clinical drugs.

Quinazoline are nitrogen-containing six-membered heterocyclic compounds that contain a benzene ring system fused to a pyrimidine at two adjacent carbon atoms. Quinazolines and their analogs possess a wide range of biological activities. Many quinazoline compounds were reported as growth factor receptor (EGFR) tyrosine kinase inhibitors, such as gefitinib, erlotinib, lapatinib and afatinib (Figure 42) [93].

**Figure 43:**
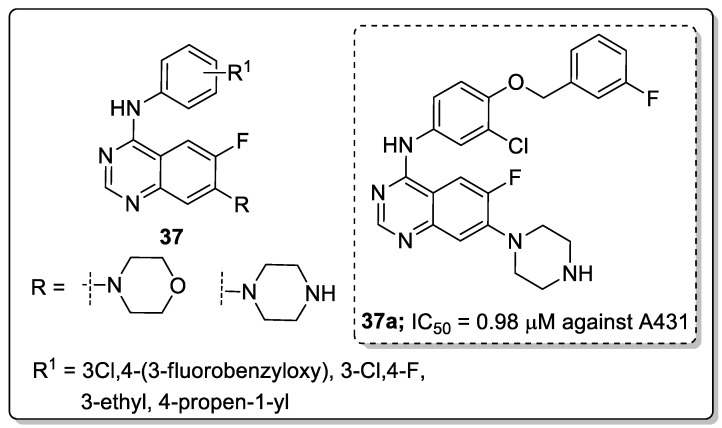
Antitumor activity of the most active quinazoline conjugate **37a**.

Quinazoline derivatives **37** were synthesized and evaluated the new derivatives for their antitumor activity [94]. Among them, compound **37a** exhibited excellent activity (IC_50_ = 0.98 μM) against the epidermoid carcinoma (A431) cell lines (Figure 43). A smaller inhibitory activity against all tested tumor cell lines (SW480, A431, A549, NCI-H1975 and HCC827) was observed when the morpholine moiety was replaced with a piperazine moiety. Compound **37a** effectively inhibited growth and metastasis in a zebrafish xenograft model. The compound induced cell apoptosis and caused cell cycle arrest at the G0/G1 phase. Furthermore, compound **37a** showed increased fluorescence intensity in a dose-dependent manner. Thus, quinazoline derivatives are promising candidates for the development of antitumor agents.

**Figure 44:**
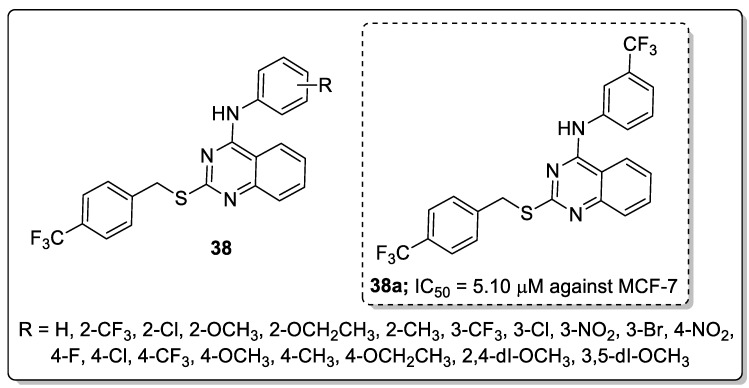
Antitumor activity of the most active quinazoline conjugate **38a**.

A library of 2,4-disubstituted-quinazoline conjugates **38** was synthesized and screened for antitumor activity against five human cancer cell lines, namely, breast (MCF-7 and MDA-MB-231), gastric carcinoma (HGC-27 and MGC-803) and prostate (PC-3) cancer cells by means of the MTT assay [95]. Among them, the trifluoromethyl derivative **38a** displayed the most promising anticancer activity (IC_50_ = 5.10 μM) against the MCF-7 breast cancer cell line when compared with the standard gefitinib (IC_50_ = 7.34 μM) (Figure 44). SAR studies revealed that electron-withdrawing groups at the *para*-position on the phenyl ring showed excellent growth inhibition activity rather than electron-donating groups. Compound **38a** caused G1 phase cell cycle arrest and cell apoptosis. Compound **38a** markedly decreased p-EGFR and p-PI3K expression, which revealed that compound **38a** targeted breast cancer cells via interference with the EGFR-PI3K signaling pathway. Furthermore, compound **38a** bound well into the active site of EGFR and formed hydrogen bonds with Lys833, Lys890 and Asp836 residues. The benzene ring of aniline interacted with Tyr867 through a π-H interaction, which indicates that compound **38a** could tightly connect with EGFR. The disubstituted-quinazoline derivatives are potential molecules for the progress of antitumor agents.

**Figure 45:**
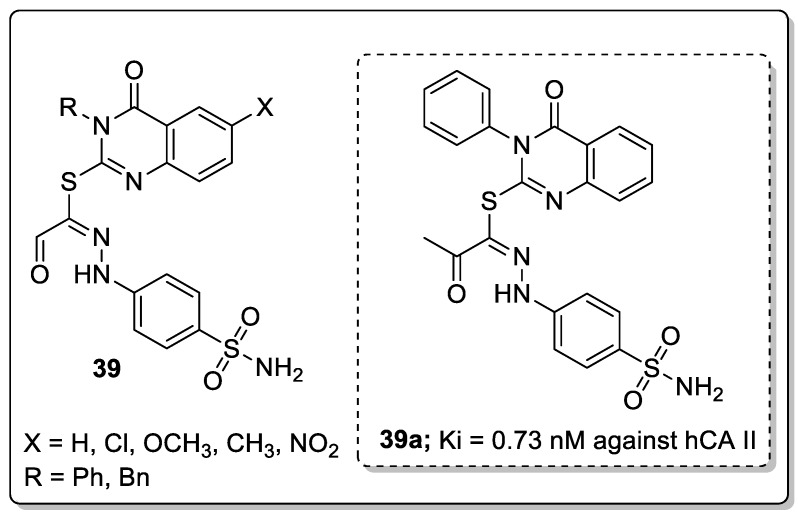
Carbonic anhydrase inhibitory activity of the most active quinazoline conjugate **39a**.

Another interesting series of quinazoline analogs **39** attached to a benzenesulfonamide was synthesized and evaluated for carbonic anhydrase inhibitory activity against the human CA (hCA) isoforms I, II, IX, and XII [96]. Among them, the unsubstituted phenyl ring compound **39a** showed significant inhibitory activity with a Ki value of 0.73 nM against hCA II. Compound **39a** was 16-fold more active than the standard acetazolamide (Ki = 12.0 nM) (Figure 45). SAR studies revealed that phenyl-substituted derivatives are more active than benzyl-substituted derivatives. Unsubstituted quinazoline derivatives showed more significant activity than those derivatives with electron-donating substituents.

**Figure 46:**
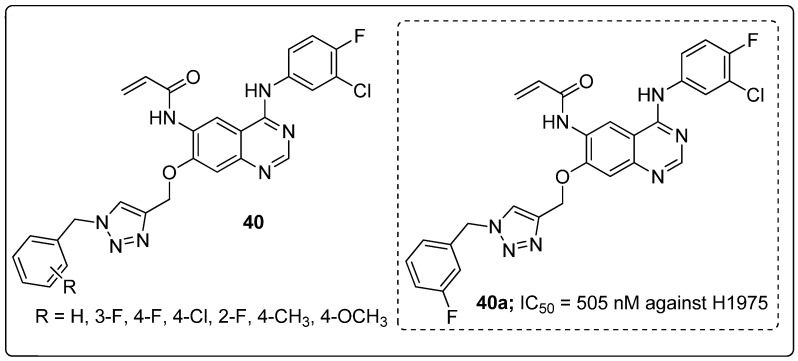
EGFR tyrosine kinase inhibitory activity of the most active quinazoline-1,2,3-triazole hybrids **40a**.

Quinazoline hybrids **40** with 1,2,3-triazole linkers were prepared and evaluated for their epidermal growth factor receptor (EGFR) tyrosine kinase inhibitory activity [97]. Compound **40a** bearing a *meta*-fluoro benzyl hybrid exhibited potent inhibitory activity (IC_50_ = 505 nM) against the H1975 cancer cell line (Figure 46). The compound exhibited 3.5-fold superior inhibitory activity against the EGFR L858R/T790M than the standard afatinib and also displayed 52- and 17-fold selectivity for EGFR L858R/T790M over wild-type HER2 and EGFR. Reduced inhibitory activity was observed in derivatives with electron-donating and electron-neutral group substituents on the benzyl ring than the fluoro-substituted compounds. In addition, compound **40a** showed moderate inhibitory activity for the Cyp450 enzyme and less toxicity for hERG and HepG2 cell lines. These results indicate that **40a** presents a low risk of cardiac arrhythmia and hepatic toxicity. The quinazoline-triazole hybrids are promising hybrids for the progress of kinase inhibitors for mutant epidermal growth factor receptors.

**Figure 47:**
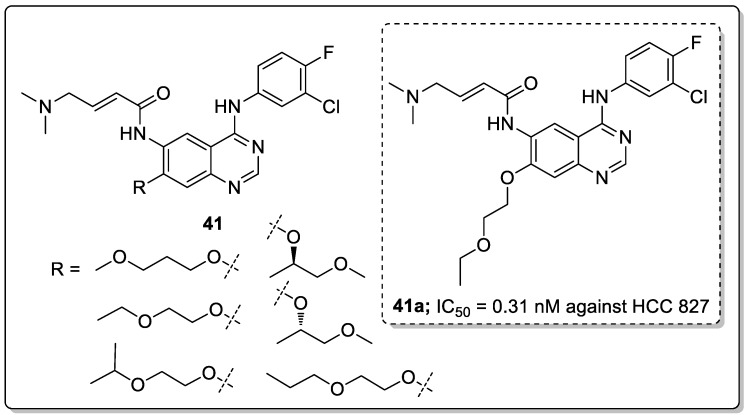
EGFR tyrosine kinase inhibitory activity of the most active quinazoline scaffold **41a**.

Quinazoline scaffolds **41** were synthesized and tested for dual EGFR/HER2 tyrosine kinase (TK) inhibitory activity [98]. Among them, compound **41a** exhibited potential inhibitory activity (IC_50_ = 0.31 nM) against the human non-small cell lung cancer cell line HCC 827 when compared with the standard afatinib (IC_50_ = 0.43 nM) (Figure 47). In addition, the active compound **41a** significantly inhibited in vitro kinase and displayed superior activities with IC_50_ values of 0.76 nM for EGFR and 39.2 nM for HER2, compared with afatinib (0.96 nM for EGFR and 73.72 nM for HER2). SAR studies revealed that the chiral variation of the alkoxy chain conjugates had poor antiproliferative inhibitory effect on H1975 and HCC827. Furthermore, in in vivo studies, compound **41a** showed a better tumor inhibition effects in nude mice NCl-H1975. Thus, the quinazoline derivatives are potential candidates for the development of cancer therapeutic agents.

### 4.3. Pyrimidines

**Figure 48:**
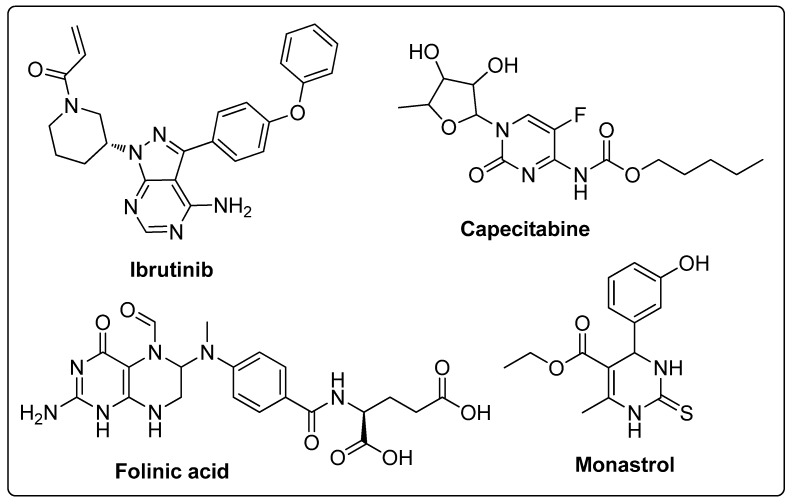
Pyrimidine and pyrimidinone clinical drugs.

Pyrimidines and pyrimidinones have received considerable attention in organic synthesis because of their wide range of biological activities. The pyrimidine nucleus consists of a six-membered 1,3-diazine ring with a ketone unit. Pyrimidine analogs are an integral part of several biologically active molecules such as natural products and nucleic acids. Moreover, this kind of heterocyclic compound finds several therapeutic applications in medicinal chemistry as an essential building block of a large variety of drug candidates and nucleic acids, with a structural resemblance to purines [99,100]. Recently, the US-FDA approved some pyrimidine and pyrimidinone derivatives (ibrutinib, capecitabine, folinic acid and monastrol) as anticancer agents (Figure 48) [18]. These pyrimidines and their scaffolds exhibit a broad spectrum of bioactivity; hence they occupy privileged positions in drug discovery studies.

**Figure 49:**
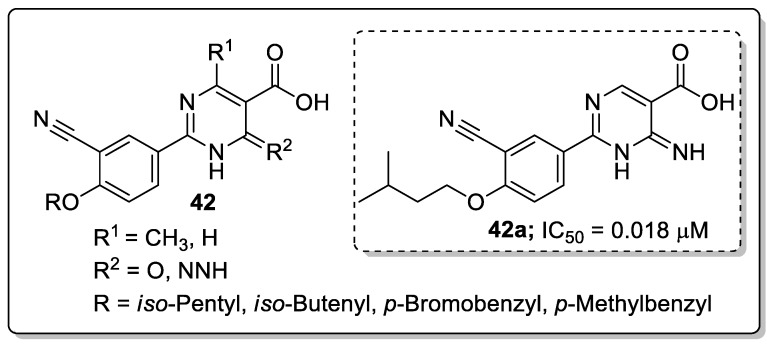
Xanthine oxidase (XO) inhibitory activity of the most active dihydropyrimidine-5-carboxylic acid analog **42a**.

Dihydropyrimidine-5-carboxylic acid analogs **42** were synthesized and evaluated for their xanthine oxidase (XO) inhibitory activity [101]. All the tested molecules exhibited good XO inhibitory activity with IC_50_ values ranging from 0.018–0.567 μM. Among them, the *iso*-butenyl derivative **42a** showed excellent inhibitory activity with an IC_50_ value of 0.018 μM, which was almost equal potency as the standard febuxostat (IC_50_ = 0.023 μM) (Figure 49). Due to amide-enamine tautomerism, the carbonyl group in the position 6 of the pyrimidine ring could function as a hydrogen bonding acceptor or donor and is linked to Thr1010 via a hydrogen bonding. The introduction of a methyl group at the 4-position of the dihydropyrimidine moiety caused a decrease in inhibition potency. Furthermore, compound **42a** was evaluated for in vivo hypouricemic effect and significantly reduced the serum uric acid (sUA) and exhibited a promising uric acid lowering property for the treatment of hyperuricemia. The active compound **42a** fitted well into the binding pocket of XO and the hydrogen bonding formed between the amino group and Glu802 was stronger than that between the carbonyl group and Thr1010 residue. Therefore, the interaction of the amino group with Glu802 should be emphasized for the design of novel nonpurine XO inhibitors.

**Figure 50:**
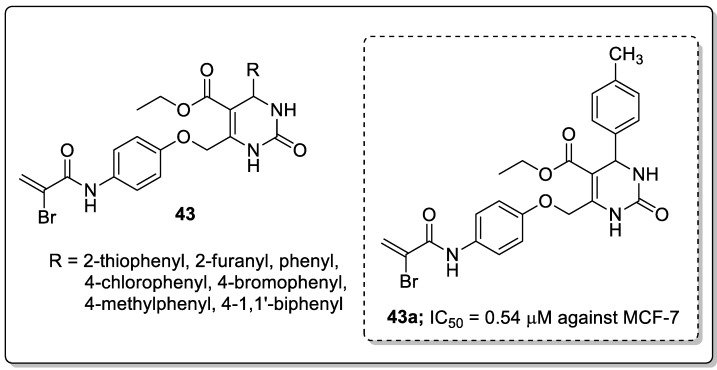
Antiproliferative activity of the most active dihydropyrimidinone conjugate **43a**.

Dihydropyrimidinone conjugates **43** were created and tested for antiproliferative and tubulin polymerization inhibitory activities [102]. Among the tested compounds, the derivative **43a** with a 4-methylphenyl ring exhibited the highest antiproliferation activity with IC_50_ values of 0.54 μM and 1.18 μM against the MCF-7 and MDA-MB-231 breast cancer cell lines (Figure 50). The compounds bearing five-membered heterocyclic thiophenyl and furanyl rings at the C4 position showed moderate cytotoxicity in comparison with derivatives with a methyl substitutent on the phenyl ring. Compound **43a** caused cell cycle arrest at the G2/M phase in a dose-dependent manner and effectively inhibited tubulin polymerization. Furthermore, compound **43a** was evaluated by molecular docking studies, and which was formed three hydrogen bonding interactions with the catalytic active site (CAS) residues Ser178, Val238 and Val318 of *α/*β-tubulin.

**Figure 51:**
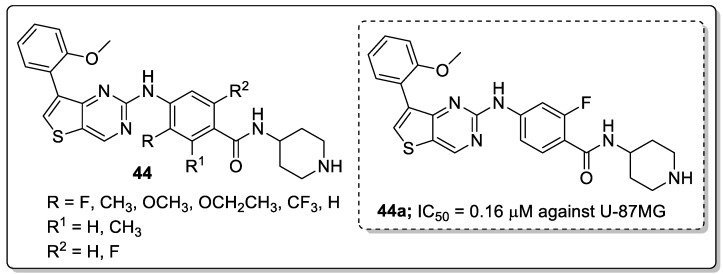
Antiproliferative activity of the most active thieno[3,2-d]pyrimidine conjugate **44a**.

Thieno[3,2-*d*]pyrimidine analogs **44** were prepared and screened for antiproliferative and focal adhesion kinase (FAK) inhibitory activities [103]. Among them, the fluoro-substituted derivative **44a** exhibited significant activity against U-87MG, A-549 and MDA-MB-231 cells with IC_50_ values of 0.16, 0.27 and 0.19 μM respectively (Figure 51). Compound **44a** also strongly inhibited the FAK enzyme (IC_50_ = 28.2 μM) and induced apoptosis in a dose-dependent manner of MDA-MB-231. Compound **44a** caused cell cycle arrest at the G0/G1 phase and showed low cytotoxicity against the normal human cell line (HK2). Furthermore, compound **44a** can be docked into the ATP-binding site of FAK where it was anchored to the hinge region via canonical donor-acceptor hydrogen-bonding motifs between the nitrogen molecules on the 2,7-disubstituted thieno[3,2-*d*]pyrimidine moiety and the backbone of residue Cys502. Further stabilization was achieved through the hydrophobic interactions of the thieno[3,2-*d*]pyrimidine ring with the hydrophobic side chains of Leu553 and Ala452 residues. Thus, the compound is a potential candidate for FAK-targeted anticancer agents.

**Figure 52:**
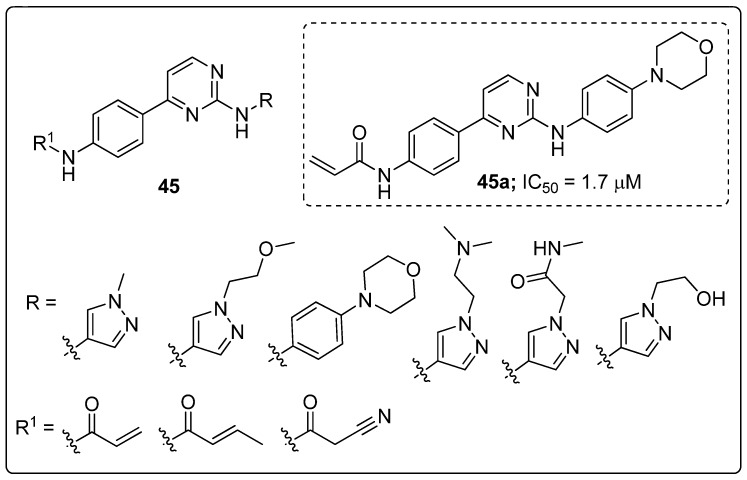
Most active pyrimidine scaffold **45a** as a JAK3 inhibitor.

Pyrimidine scaffolds **45** were synthesized and evaluated the scaffolds for Janus kinase 3 (JAK3) inhibitory activity [104]. Among them compound **45a** with a morpholine unit attached to the phenyl ring displayed the highest JAK3 inhibitory activity with an IC_50_ value of 1.7 μM, compared with the standard tofacitinib (IC_50_ = 0.9 μM) (Figure 52). The 4-morpholinoaniline exhibited a higher potency than a pyrazole ring at the 2-position of the pyrimidine ring. Compound **45a** strongly inhibited T cell proliferation (IC_50_ = 0.83 μM) and high selectivity within the JAK family. Moreover, in vivo data revealed that compound **45a** significantly suppressed oxazolone (OXZ)-induced delayed hyper-sensitivity responses in Balb/c mice. The compound showed good oral pharmacokinetic properties. Furthermore, the molecular docking study of compound **45a**, revealed that the hydrophobic aromatic amine group showed *σ*-*π* interactions with the amino acid Leu828 and Gly908 residues, and the bidentate hinge hydrogen bonding formed with the Leu905 residue in the active sites of JAK3. Therefore, the compound is a potential candidate as a selective JAK3 inhibitor for treating autoimmune diseases.

**Figure 53:**
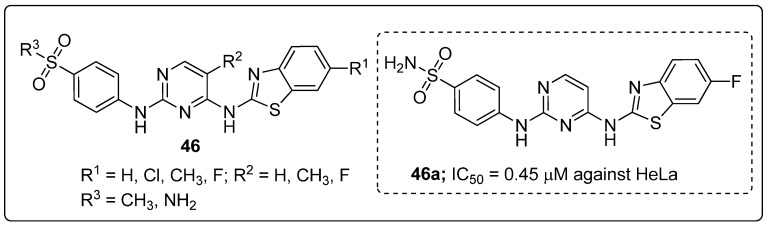
Anticancer activity of the most active pyrimidine-benzothiazole hybrid **46a**.

Pyrimidines **46** tethered to benzothiazole hybrids were designed and screened for anticancer activity against five cancer cell lines (HCT116, MCF-7, MDA-MB-231, HeLa and PC-3) [105]. The hybrid compound **46a** bearning a fluoro group exhibited highest anticancer activity with IC_50_ values of 0.70 μM and 0.45 μM against the HCT116 and HeLa cell lines, respectively (Figure 53). The compound **46a** also exhibited 3-fold stronger CDK2 inhibitory potency (IC_50_ = 15.4 nM) than the standard AZD5438 (IC_50_ = 45 nM). Replacement of the methyl group with a fluoro atom in the pyrimidine hybrid resulted in reduced potency against both HeLa and HCT116 cells. Compound **46a** effectively induced apoptosis and cell cycle arrest at the G2/M phase in a concentration-dependent manner. Furthermore, the active compound **46a** exhibited effective binding into the CDK2 binding site. The oxygen and nitrogen atoms of the sulfonyl moiety interacted with the Asp86 residue through hydrogen bonding. Moreover, the Leu83 residue formed hydrogen bonding with the 2-amino moiety and nitrogen on the pyrimidine ring. Another important hydrogen bonding was detected between the sulfur atom on the benzothiazole ring and the residue Lys33. These pyrimidine hybrids are potential analogs as a cyclin-dependent kinase 2 (CDK2) inhibitors.

**Figure 54:**
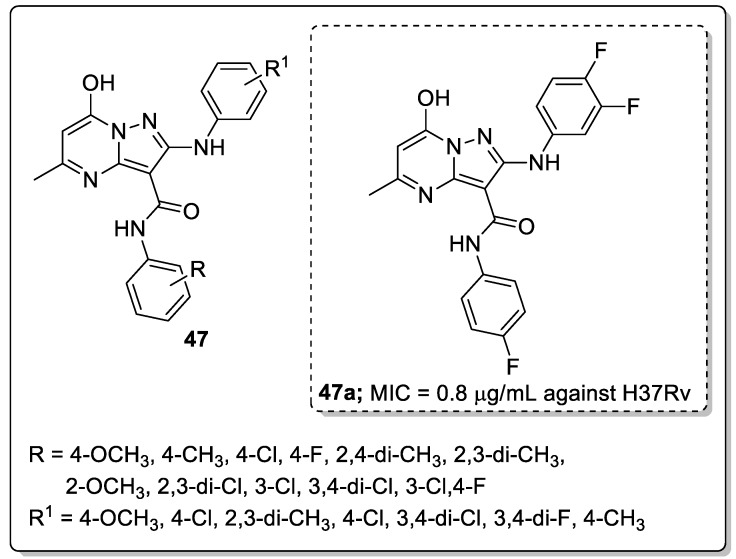
The antitubercular activity of the most active pyrimidine -fused pyrazole hybrid **47a**.

Pyrimidine fused pyrazole hybrids **47** were synthesized and tested for their anti-tubercular activity against the H37Rv strain [106]. All the tested hybrids showed good to excellent anti-tubercular activity with MIC values ranging from 0.8 to 50 μg/mL (Figure 54). Among them, the fluoro-substituted hybrid **47a** exhibited the most potent anti-tubercular activity, with a MIC value of 0.8 μg/mL, which was almost 4-fold stronger potency than the standard ciprofloxacin (MIC = 3.12 μg/mL). SAR studies revealed that electron-donating groups showed reduced the activity in comparison with halogen-substituted derivatives. Furthermore, the active compound **47a** displayed no cytotoxicity and the H-bonding interactions with Lys165, Tyr196, and Ile194 through the water molecule, contributed to the binding affinity of the ligand. These compounds are promising hybrids for the development of novel anti-tubercular agents.

**Figure 55:**
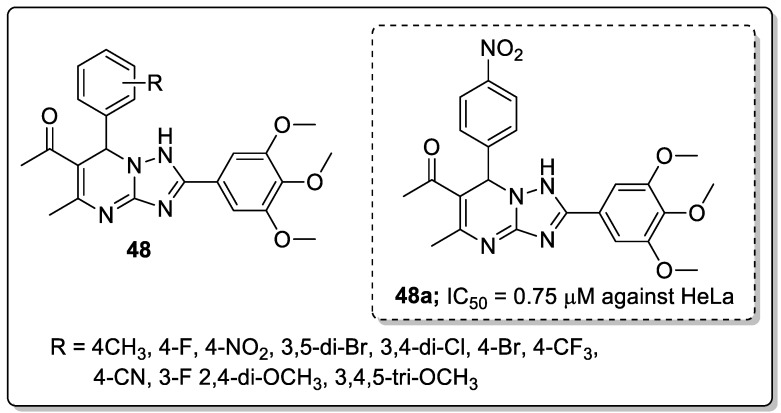
Antiproliferative activity of the most active 1,2,4-triazole-pyrimidine hybrid **48a**.

Pyrimidine hybrids **48** with a 1,2,4-triazole linker were created and tested for antiproliferative activity against four human alveolar epithelial (A549), cervical (HeLa), colon (HCT116) and embryonic kidney (HEK293) cancer cells [107]. Compound **48a** bearing a *para*-nitro group on the phenyl ring exhibited the most significant antiproliferative activity against A549 and HeLa cells with IC_50_ values of 1.02 μM and 0.75 μM, respectively (Figure 55). Electron-withdrawing groups, such as 4-nitro, 3,5-dibromo, 3,4-dichloro, 4-bromo, and 3-fluoro on the phenyl ring hybrids showed more activity than compounds with electron-donating groups (2,4-dimethoxy, 4-dimethoxy, 3,4,5-trimethoxy, 4-ethyl, and 3,4-dimethoxy). Compound **48a** caused cell cycle arrest at the G2/M phase. Furthermore, molecular docking studies of the active compound **48a** showed that it occupied the colchicine-binding site of tubulin. The oxygen of the 4-nitrophenyl ring established a hydrogen bonding interaction with *β*Cys241 and one nitrogen atom of the triazole ring forms a hydrogen bonding with *β*Asn258, and the residue of *α*Thr178 forms a hydrogen bonding with the oxygen of the trimethoxy group. These hybrids are potential candidates for the development of anticancer agents.

**Figure 56:**
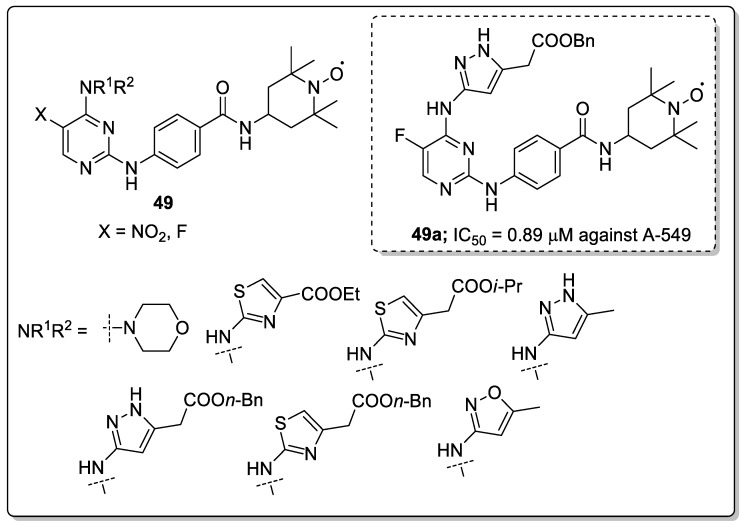
Antiproliferative activity of the most potent pyrimidine-linked nitroxide derivative **49a**.

Pyrimidine-linked nitroxide derivatives **49** were synthesized and screened for anti-proliferative and Aurora kinases inhibitory activities [108]. Among the series, the butyl- and fluoro-substituted compound **49a** exhibited the most significant activity, with an IC_50_ value of 0.89 μM against the lung carcinoma (A-549) cancer cell line (Figure 56). Compound **49a** also exhibited kinase inhibitory activity against Aurora-A (IC_50_ = 9.3 μM) and Aurora-B (IC_50_ = 2.8 μM) in HeLa cells. In addition, compound **49a** effectively inhibited the phosphorylation of HisH3 and reduced the expression of protein Eg5 and TPX2 in a dose-dependent manner. Furthermore, compound **49a** bound well with Aurora-A and Aurora-B. For Aurora-A, the pyrimidine ring of **49a** occupied the adenine-binding region and formed an essential hydrogen-bonding interaction with Ala213 in the hinge region. For Aurora-B, the two nitrogen atoms of the pyrazole group in **49a** formed hydrogen-bonding interactions with the key amino acid residues Glu155 and Ala157 of the hinge region and the amide bond at C-2 of the pyrimidine form hydrogen-bonding interaction with Pro158 residues. The pyrimidine-linked nitroxide analogs are potent molecules for the development of anticancer agents as Aurora kinase inhibitors.

## 5. Conclusions

The scope of nitrogen-based compounds in medicine is growing daily and their diverse analogs provide a viable and important path for the discover of drugs with various biological applications. The *N*-heterocyclic frameworks offer a high degree of structural diversity that has proven useful for the search of new therapeutic agents in improving the pharmacokinetics and other physicochemical features. Numerous drugs that are currently in clinical practice have fatal side-effects and have developed multidrug resistance, and have been extensively used in practice to treat various types of diseases with high therapeutic potency. Research and development of nitrogen-based compounds in medicinal chemistry has become a rapidly developing and increasingly active topic. A large amount of work has been made towards *N*-heterocyclic skeleton medicinal chemistry. The overwhelming advantages of nitrogen-containing drugs in the medicinal field, including easy preparation, low toxicity, less adverse effects, high bioavailability, lower drug resistance, good biocompatibility, etc., encourage efforts towards further research and development. Hence, the properties of these scaffolds are vital to the synthetic strategy in the current drug discovery and design system. In this review, we mainly covered and widely described the current trends in families of nitrogen-based heterocyclic molecules namely, β-lactam, pyrazole, imidazole, 1,2,4-triazole, pyrimidine, quinoline and quinazoline derivatives, with highly promising biological properties such as anticancer, anti-inflammatory, antibacterial, antifungal, antitubercular, antidiabetic, antioxidant, anti-HIV and other medicinal properties. In addition, we have explored their structure-activity relationships, as well as a binding mode through molecular docking studies. The SAR studies of the discussed molecules offered a greater understanding of the pattern of substituents on their basic skeleton and appropriate substitutions accountable for its effectiveness. These significant points confirm the enormous potential of various *N*-heterocyclic cores in pharmaceutical applications suggesting a massive scope for these promising moieties because of their diverse molecular targets. We believe that this review article will be valuable for encouraging the structural design and development of sustainable and effective nitrogen-based drugs against various diseases, with minimal side-effects.

## Figures and Tables

**Table 1:** List of nitrogen-containing clinical drugs.

S. No	Structure	Drug Name	Activity
**1**	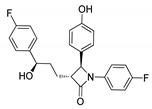	Ezetimibe	Cholesterol absorption inhibitor [27]
**2**	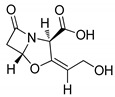	Clavulanic acid	β-Lactamase inhibitor [28]
**3**	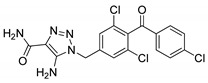	Carboxyamidotriazole	Calcium channel blocker as an anti-cancer [29]
**4**	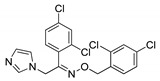	Oxiconazole	Antifungal [30]
**5**	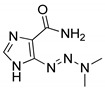	Dacarbazine	Treatment of metastatic melanoma [31]
**6**	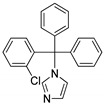	Clotrimazole	Antifungal [31]
**7**	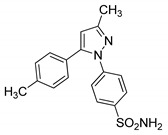	Celecoxib	Anti-inflammatory [32]
**8**	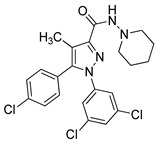	Rimonabant	Anti-obesity [33]
**9**	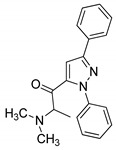	Difenamizole	Anti-analgesic [34]
**10**	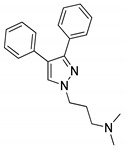	Fezolamine	Antidepressant [35]
**11**	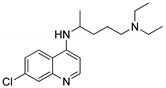	Chloroquine	Antimalarial [36]
**12**	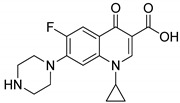	Ciprofloxacin	Antibiotic [36]
**13**	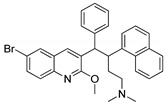	Bedaquiline	Anti-TB [37]
**14**	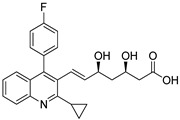	Pitavastatin	Cholesterol-lowering agent [37]
**15**	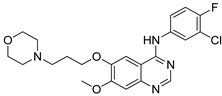	Gefitinib	Growth factor receptor (EGFR) tyrosine kinase inhibitor [38]
**16**	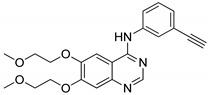	Erlotinib	Treating metastaticnon-small-cell lung cancer (NSCLC) [38]
**17**	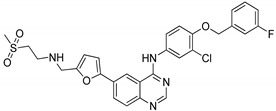	Lapatinib	Anti-breast cancer [39]
**18**	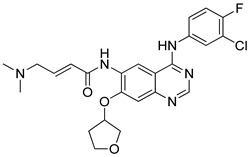	Afatinib	Irreversible covalent inhibitorof the receptor tyrosine kinases (RTK) for EGFR [39]
**19**	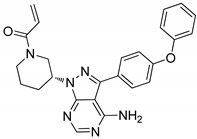	Ibrutinib	Chronic lymphocytic leukemia cancer [18]
**20**	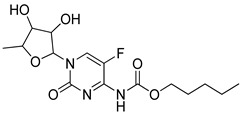	Capecitabine	Ant-breast cancer [18]
**21**	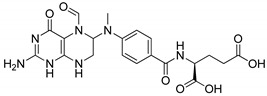	Folinic acid	Anti-colorectal cancer [40]
**22**	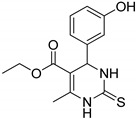	Monastrol	Inhibitor of mitotic spindle protein [40]

## References

[1] Li X., He L., Chen H., Wu W., Jiang H. (2013). Copper-catalyzed aerobic C(sp^2^)–H functionalization for C–N bond formation: Synthesis of pyrazoles and indazoles. J. Org. Chem..

[2] Santos C.M.M., Freitas M., Fernandes E. (2018). A comprehensive review on xanthone derivatives as α-glucosidase inhibitors. Eur. J. Med. Chem..

[3] Kalaria P.N., Karad S.C., Raval D.K. (2018). A review on diverse heterocyclic compounds as the privileged scaffolds in antimalarial drug discovery. Eur. J. Med. Chem..

[4] Kerru N., Bhaskaruni S.V.H.S., Gummidi L., Maddila S.N., Maddila S., Jonnalagadda S.B. (2019). Recent advances in heterogeneous catalysts for the synthesis of imidazole derivatives. Synth. Commun..

[5] Kerru N., Singh P., Koorbanally N., Raj R., Kumar V. (2017). Recent advances (2015–2016) in anticancer hybrids. Eur. J. Med. Chem..

[6] Eftekhari-Sis B., Zirak M., Akbari A. (2013). Arylglyoxals in synthesis of heterocyclic compounds. Chem. Rev..

[7] Kerru N., Maddila S., Jonnalagadda S.B. (2019). Design of carbon–carbon and carbon–heteroatom bond formation reactions under green conditions. Curr. Org. Chem..

[8] Ju Y., Varma R.S. (2006). Aqueous *N*-heterocyclization of primary amines and hydrazines with dihalides:  microwave-assisted syntheses of *N*-azacycloalkanes, isoindole, pyrazole, pyrazolidine, and phthalazine derivatives. J. Org. Chem..

[9] Zarate D.Z., Aguilar R., Hernandez-Benitez R.I., Labarrios E.M., Delgado F., Tamariz J. (2015). Synthesis of α-ketols by functionalization of captodative alkenes and divergent preparation of heterocycles and natural products. Tetrahedron.

[10] Leeson P.D., Springthorpe B. (2007). The influence of drug-like concepts on decision-making in medicinal chemistry. Nat. Rev. Drug Discov..

[11] Fang W.Y., Ravindar L., Rakesh K.P., Manukumar H.M., Shantharam C.S., Alharbi N.S., Qin H.L. (2019). Synthetic approaches and pharmaceutical applications of chloro-containing molecules for drug discovery: A critical review. Eur. J. Med. Chem..

[12] Kerru N., Singh-Pillay A., Awolade P., Singh P. (2018). Current anti-diabetic agents and their molecular targets: A review. Eur. J. Med. Chem..

[13] Smith B.R., Eastman C.M., Njardarson J.T. (2014). Beyond C, H, O, and N analysis of the elemental composition of U.S. FDA approved drug architectures. J. Med. Chem..

[14] Vitaku E., Smith D.T., Njardarson J.T. (2014). Analysis of the structural diversity, substitution patterns, and frequency of nitrogen heterocycles among U.S. FDA approved Pharmaceuticals. J. Med. Chem..

[15] Gordon E.M., Barrett R.W., Dower W.J., Fodor S.P.A., Gallop M.A. (1994). Applications of combinatorial technologies to drug discovery, combinatorial organic synthesis, library screening strategies, and future directions. J. Med. Chem..

[16] Walsh C.T. (2015). Nature loves nitrogen heterocycles. Tetrahedron Lett..

[17] Zhang B., Studer A. (2015). Recent advances in the synthesis of nitrogen heterocycles via radical cascade reactions using isonitriles as radical acceptors. Chem. Soc. Rev..

[18] Kaur R., Chaudhary S., Kumar K., Gupta M.K., Rawal R.K. (2017). Recent synthetic and medicinal perspectives of dihydropyrimidinones: A review. Eur. J. Med. Chem..

[19] Chaudhari K., Surana S., Jain P., Patel H.M. (2016). Mycobacterium tuberculosis (MTB) GyrB inhibitors: An attractive approach for developing novel drugs against TB. Eur. J. Med. Chem..

[20] Sameem B., Saeedi M., Mahdavi M., Shafiee A. (2017). A review on tacrine-based scaffolds as multi-target drugs (MTDLs) for Alzheimer’s disease. Eur. J. Med. Chem..

[21] Akhtar J., Khan A.A., Ali Z., Haider R., Yar M.S. (2017). Structure-activity relationship (SAR) study and design strategies of nitrogen-containing heterocyclic moieties for their anticancer activities. Eur. J. Med. Chem..

[22] Ma X., Lv X., Zhang J. (2018). Exploiting polypharmacology for improving therapeutic outcome of kinase inhibitors (KIs): An update of recent medicinal chemistry efforts. Eur. J. Med. Chem..

[23] Kaur R., Dahiya L., Kumar M. (2017). Fructose-1,6-bisphosphatase inhibitors: A new valid approach for management of type 2 diabetes mellitus. Eur. J. Med. Chem..

[24] Patel R.V., Keum Y.S., Park S.W. (2015). Sketching the historical development of pyrimidones as the inhibitors of the HIV integrase. Eur. J. Med. Chem..

[25] Martins P., Jesus J., Santos S., Raposo L.R., Rodrigues C.R., Baptista P.V., Fernandes A.R. (2015). Heterocyclic anticancer compounds: Recent advances and the paradigm shift towards the use of nanomedicine’s toolbox. Molecules.

[26] Nitrogen Heterocycles in Medicinal Chemistry. https://www.scopus.com/sources.uri.

[27] Arya N., Jagdale A.Y., Patil T.A., Yeramwar S.S., Holikatti S.S., Dwivedi J., Shishoo C.J., Jain K.S. (2014). The chemistry and biological potential of azetidin-2-ones. Eur. J. Med. Chem..

[28] Singh G.S., Sudheesh S. (2014). Advances in synthesis of monocyclic β-lactams. Arkivoc.

[29] Xu Z., Zhao S.J., Liu Y. (2019). 1,2,3-Triazole-containing hybrids as potential anticancer agents: Current developments, action mechanisms and structure-activity relationships. Eur. J. Med. Chem..

[30] Liu Z., Zhang Z., Zhang W., Yan D. (2018). 2-Substituted-1-(2-morpholinoethyl)-1*H*-naphtho[2,3-d]imidazole-4,9-diones: Design, synthesis and antiproliferative activity. Bioorg. Med. Chem. Lett..

[31] Zhang L., Peng X.M., Damu G.L.V., Geng R.X., Zhou C.H. (2014). Comprehensive review in current developments of imidazole-based medicinal chemistry. Med. Res. Rev..

[32] Baumann M., Baxendale I.R., Ley S.V., Nikbin N. (2011). An overview of the key routes to the bestselling 5-membered ring heterocyclic pharmaceuticals. Beilstein J. Org. Chem..

[33] Karrouchi K., Radi S., Ramli Y., Taoufik J., Mabkhot Y.N., Al-Aizari F.A., Ansar M. (2018). Synthesis and pharmacological activities of pyrazole derivatives: A Review. Molecules.

[34] Silva V.L.M., Elguero J., Silva A.M.S. (2018). Current progress on antioxidants incorporating the pyrazole core. Eur. J. Med. Chem..

[35] Ansari A., Ali A., Asif M. (2017). Review: Biologically active pyrazole derivatives. New J. Chem..

[36] Jain S., Chandra V., Jain P.K., Pathak K., Pathak D., Vaidya A. (2019). Comprehensive review on current developments of quinoline-based anticancer agents. Arabian J. Chem..

[37] Zhang J., Wang S., Ba Y., Xu Z. (2019). 2,4-Triazole-quinoline/quinolone hybrids as potential anti-bacterial agents. Eur. J. Med. Chem..

[38] Ahmad I. (2017). An insight into the therapeutic potential of quinazoline derivatives as anticancer agents. Med. Chem. Commun..

[39] Alagarsamy V., Chitra K., Saravanan G., Solomon V.R., Sulthana M.T., Narendhar B. (2018). An overview of quinazolines: Pharmacological significance and recent developments. Eur. J. Med. Chem..

[40] Vendrusculo V., de Souza V.P., Fontour L.A.M., D’Oca M.G., Banzato T.P., Monteiro P.A., Pilli R.A., de Carvalho J.E., Russowsky D. (2018). Synthesis of novel perillyl-dihydropyrimidinone hybrids designed for antiproliferative activity. Med. Chem. Commun..

[41] Newman D.J., Cragg G.M. (2016). Natural products as sources of new drugs from 1981 to 2014. J. Nat. Prod..

[42] Robb M.J., Moore J.S. (2015). A retro-staudinger cycloaddition: Mechanochemical cycloelimination of a β-lactam mechanophore. J. Am. Chem. Soc..

[43] Baiula M., Galletti P., Martelli G., Soldati R., Belvisi L., Civera M. (2016). New β-lactam derivatives modulate cell adhesion and signaling mediated by RGD-binding and leukocyte integrins. J. Med. Chem..

[44] Majewski M.W., Miller P.A., Oliver A.G., Miller M.J. (2017). Alternate “drug” delivery utilizing β-lactam cores: Syntheses and biological evaluation of β-lactams bearing isocyanate precursors. J. Org. Chem..

[45] Cele Z.E.D., Arvidsson P.I., Kruger H.G., Govender T., Naicker T. (2015). Applied enantioselective aminocatalysis: α-Heteroatom functionalization reactions on the carbapenem (β-lactam antibiotic) core. Eur. J. Org. Chem..

[46] Wang Y., Zhang H., Huang W., Kong J., Zhou J., Zhang B. (2009). 2-Azetidinone derivatives: Design, synthesis and evaluation of cholesterol absorption inhibitors. Eur. J. Med. Chem..

[47] Kamath A., Ojima I. (2012). Advances in the chemistry of β-lactam and its medicinal applications. Tetrahedron.

[48] Hosseyni S., Jarrahpour A. (2018). Recent advances in β-lactam synthesis. Org. Biomol. Chem..

[49] Han W.T., Trehan A.K., Wright J.J.K., Federici M.E., Seiler S.M., Meanwell N.A. (1995). Azetidin-2-one derivatives as inhibitors of thrombin. Bioorg. Med. Chem..

[50] Borazjani N., Sepehri S., Behzadi M., Jarrahpour A., Rad J.A., Sasanipour M., Mohkam M., Ghasemi Y., Akbarizadeh A.R., Digiorgio C. (2019). Three-component synthesis of chromeno β-lactam hybrids for inflammation and cancer screening. Eur. J. Med. Chem..

[51] Malebari A.M., Darren F., Nathwani S.M., O’Connell F., Noorani S., Twamley B., O’Boyle N.M., O’Sullivan J., Zisterer D.M., Meegan M.J. (2020). β-Lactams with antiproliferative and antiapoptotic activity in breast and chemoresistant colon cancer cells. Eur. J. Med. Chem..

[52] Mohamadzadeha M., Zareib M., Vessala M. (2020). Synthesis, in vitro biological evaluation and in silico molecular docking studies of novel β-lactam-anthraquinone hybrids. Bioorg. Chem..

[53] Kuskovsky R., Lloyd D., Arora K., Plotkin B.J., Green J.M., Boshoff H.I., Barry C., Deschamps J., Konaklieva M.I. (2019). C4-Phenylthio β-lactams: Effect of the chirality of the β-lactam ring on antimicrobial activity. Bioorg. Med. Chem..

[54] Bozorov K., Zhao J., Aisa H.A. (2019). 1,2,3-Triazole-containing hybrids as leads in medicinal chemistry: A recent overview. Bioorg. Med. Chem..

[55] Dheer D., Singh V., Shankar R. (2017). Medicinal attributes of 1,2,3-triazoles: Current developments. Bioorg. Chem..

[56] Qiana J., Hana Y., Lia J., Zhang J., Hu C. (2018). Toxic effect prediction of cefatirizine amidine sodium and its impurities by structure-toxicity relationship of cephalosporins. Toxicol. Vitro.

[57] Reddyrajula R., Dalimba U., Madan K.S. (2019). Molecular hybridization approach for phenothiazine incorporated 1,2,3-triazole hybrids as promising antimicrobial agents: Design, synthesis, molecular docking and in silico ADME studies. Eur. J. Med. Chem..

[58] Sun L., Huang T., Dick A., Meuser M.E., Zalloum W.A., Chen C.H., Ding X., Gao P., Cocklin S., Lee K.H. (2020). Design, synthesis and structure-activity relationships of 4-phenyl-1H-1,2,3-triazole phenylalanine derivatives as novel HIV-1 capsid inhibitors with promising antiviral activities. Eur. J. Med. Chem..

[59] Fu D.J., Li P., Wu B.W., Cui X.X., Zhao C.B., Zhang S.Y. (2019). Molecular diversity of trimethoxyphenyl-1,2,3-triazole hybrids as novel colchicine site tubulin polymerization inhibitors. Eur. J. Med. Chem..

[60] Ashour H.F., Abou-Zeid L.A., El-Sayed M.A.A., Selim K.B. (2020). 1,2,3-Triazole-Chalcone hybrids: Synthesis, in vitro cytotoxic activity and mechanistic investigation of apoptosis induction in multiple myeloma RPMI-8226. Eur. J. Med. Chem..

[61] Asgari M.S., Mohammadi-Khanaposhtani M., Kiani M., Ranjbar P.R., Zabihi E., Pourbagher R., Rahimi R., Faramarzi M.A., Biglar M., Larijani B. (2019). Biscoumarin-1,2,3-triazole hybrids as novel anti-diabetic agents: Design, synthesis, in vitro α-glucosidase inhibition, kinetic, and docking studies. Bioorg. Chem..

[62] Saeedi M., Mohammadi-Khanaposhtani M., Pourrabi P., Razzaghi N., Ghadimi R., Imanparast S., Faramarzi M.A., Bandarian F., Esfahani E.N., Safavi M. (2019). Design and synthesis of novel quinazolinone-1,2,3-triazole hybrids as new anti-diabetic agents: In vitro *α*-glucosidase inhibition, kinetic, and docking study. Bioorg. Chem..

[63] Saeedi M., Mohammadi-Khanaposhtani M., Asgari M.S., Eghbalnej N., Imanparast S., Faramarzi M.A., Larijani B., Mahdavi M., Akbarz T. (2019). Design, synthesis, *in vitro*, and in silico studies of novel diarylimidazole-1,2,3-triazole hybrids as potent α-glucosidase inhibitors. Bioorg. Med. Chem..

[64] Gholampour M., Ranjbar S., Edraki N., Mohabbati M., Firuzi O., Khoshneviszadeh M. (2019). Click chemistry-assisted synthesis of novel aminonaphthoquinone-1,2,3-triazole hybrids and investigation of their cytotoxicity and cancer cell cyclealterations. Bioorg. Chem..

[65] Adib M., Peytam F., Shourgeshty R., Mohammadi-Khanaposhtani M., Jahani M., Imanparast S., Faramarzi M.A., Larijani B., Moghadamni A.A., Esfahani E.N. (2019). Design and synthesis of new fused carbazole-imidazole derivatives as antidiabetic agents: In vitro *α*-glucosidase inhibition, kinetic, and in silico studies. Bioorg. Med. Chem. Lett..

[66] Bolousa M., Arumugam N., Almansour A.I., Kumar R.S., Maruok K., Antharam V.C., Thangamani S. (2019). Broad-spectrum antifungal activity of spirooxindolo-pyrrolidine tethered indole/imidazole hybrid heterocycles against fungal pathogens. Bioorg. Med. Chem. Lett..

[67] Fan C., Zhong T., Yang H., Yang Y., Wang D., Yang X., Xu Y., Fan Y. (2020). Design, synthesis, biological evaluation of 6-(2-amino-1*H*-benzo[*d*]imidazole-6-yl)quinazolin-4(3*H*)-one derivatives as novel anticancer agents with Aurora kinase inhibition. Eur. J. Med. Chem..

[68] Li L., Quan D., Chen J., Ding J., Zhao J., Lv L., Chen J. (2019). Design, synthesis, and biological evaluation of 1-substituted-2-aryl imidazoles targeting tubulin polymerization as potential anticancer agents. Eur. J. Med. Chem..

[69] Zhang L., Ge Y., Wang Q.M., Zhou C.H. (2019). Identification of novel imidazole flavonoids as potent and selective inhibitors of protein tyrosine phosphatase. Bioorg. Chem..

[70] Guo Z., Song X., Zhao L.M., Piao M.G., Quan J., Piao H.R., Jin C.H. (2019). Synthesis and biological evaluation of novel benzo[*c*][1,2,5]thiadiazol-5-yl and thieno[3,2-*c*]-pyridin-2-yl imidazole derivatives as ALK5 inhibitors. Bioorg. Med. Chem. Lett..

[71] Ding H.W., Yu L., Bai M., Qin X.C., Song M., Zhao Q.C. (2019). Design, synthesis and evaluation of some 1,6-disubstituted-*1H*-benzo[*d*]imidazoles derivatives targeted PI3K as anticancer agents. Bioorg. Chem..

[72] Chaudhry F., Naureen S., Ashraf M., Al-Rashid M., Jahan B., Munawar M.A., Khana M.A. (2019). Imidazole-pyrazole hybrids: Synthesis, characterization and *in-vitro* bio evaluation against *α*-glucosidase enzyme with molecular docking studies. Bioorg. Chem..

[73] Tageldin G.N., Ibrahim T.M., Fahmy S.M., Ashour H.M., Khalil M.A., Nassra R.A., Labout I.M. (2019). Synthesis, modeling and biological evaluation of some pyrazolo[3,4-d] pyrimidinones and pyrazolo[4,3-e][1,2,4]triazolo[4,3-a]pyrimidinones as anti-inflammatory agents. Bioorg. Chem..

[74] Gedawy E.M., Kassab A.E., Kerdawy A.M.E. (2020). Design, synthesis and biological evaluation of novel pyrazole sulfonamide derivatives as dual COX-2/5-LOX inhibitors. Eur. J. Med. Chem..

[75] Zhu W.J., Cui B.W., Wang H.M., Nan J.X., Piao H.R., Lian L.H., Jin C.H. (2019). Design, synthesis, and antifibrosis evaluation of 4-(benzo-[c][1,2,5]thiadiazol-5-yl)-3(5)-(6-methyl- pyridin-2-yl)pyrazole and 3(5)-(6-methylpyridin-2-yl)-4-(thieno-[3,2,-c]pyridin-2-yl)pyrazole derivatives. Eur. J. Med. Chem..

[76] Abdellatif K.R.A., Fadaly W.A.A., Mostaf Y.A., Zaher D.M., Omar H.A. (2019). Thiohydantoin derivatives incorporating a pyrazole core: Design, synthesis and biological evaluation as dual inhibitors of topoisomerase-I and cycloxygenase-2 with anti-cancer and anti-inflammatory activities. Bioorg. Chem..

[77] Nagaraju B., Kovvuri J., Kumar C.G., Routhu S.R., Shareef M.A., Kadagathura M., Adiyala P.R., Alavala S., Nagesh N., Kamal A. (2019). Synthesis and biological evaluation of pyrazole linked benzothiazole-β-naphthol derivatives as topoisomerase I inhibitors with DNA binding ability. Bioorg. Med. Chem..

[78] Takate S.J., Shinde A.D., Karale B.K., Akolkar H., Nawale L., Sarkar D., Mhaske P.C. (2019). Thiazolyl-pyrazole derivatives as potential antimycobacterial agents. Bioorg. Med. Chem. Lett..

[79] Sun L., Wang P., Xu L., Gao L., Li J., Piao H. (2019). Discovery of 1,3-diphenyl-1*H*-pyrazole derivatives containing rhodanine-3-alkanoic acid groups as potential PTP1B inhibitors. Bioorg. Med. Chem. Lett..

[80] Zhao B., Liang Q., Ren H., Zhang X., Wu Y., Zhang K., Ma L.Y., Zheng Y.C., Liu H.M. (2020). Discovery of pyrazole derivatives as cellular active inhibitors of histone lysine specific demethylase 5B (KDM5B/JARID1B). Eur. J. Med. Chem..

[81] Brullo C., Massa M., Rapetti F., Alfei S., Bertolotto M.B., Montecucco F., Signorello M.G., Bruno O. (2020). New hybrid pyrazole and imidazopyrazole antinflammatory agents able to reduce ROS production in different biological targets. Molecules.

[82] Chu X.M., Wang C., Liu W., Liang L.L., Gong K.K., Zhao C.Y., Sun K.L. (2019). Quinoline and quinolone dimers and their biological activities: An overview. Eur. J. Med. Chem..

[83] Nainwal L.M., Tasneem S., Akhtar W., Verma G., Khan M.F., Parvez S., Shaquiquzzaman M., Akhter M., Alam M.M. (2019). Green recipes to quinoline: A review. Eur. J. Med. Chem..

[84] Bharate J.B., Vishwakarma R.A., Bharate S.B. (2015). Metal-free domino one-pot protocols for quinoline synthesis. RSC Adv..

[85] Su T., Zhu J., Sun R., Zhang H., Huang Q., Zhang X., Du R., Qiu L., Cao R. (2019). Design, synthesis and biological evaluation of new quinoline derivatives as potential antitumor agents. Eur. J. Med. Chem..

[86] Li S., Hu L., Li J., Zhu J., Zeng F., Huang Q., Qiu L., Du R., Cao R. (2019). Design, synthesis, structure-activity relationships and mechanism of action of new quinoline derivatives as potential antitumor agents. Eur. J. Med. Chem..

[87] Jafari F., Baghayi H., Lavaee P., Hadizadeh F., Soltani F., Moallemzadeh H., Mirzaei S., Aboutorabzadeh S.M., Ghodsi R. (2019). Design, synthesis and biological evaluation of novel benzo- and tetrahydrobenzo-[*H*]quinoline derivatives as potential DNA intercalating antitumor agents. Eur. J. Med. Chem..

[88] Li W., Shuai W., Sun H., Xu F., Bi Y., Xu J., Ma C., Yao H., Zhu Z., Xu S. (2019). Design, synthesis and biological evaluation of quinoline-indole derivatives as anti-tubulin agents targeting the colchicine binding site. Eur. J. Med. Chem..

[89] Ramprasad J., Sthalam V.K., Thampunuri R.L.M., Bhuky S., Ummanni R., Balasubramanian S., Pabbaraja S. (2019). Synthesis and evaluation of a novel quinoline-triazole analogs for antitubercular properties via molecular hybridization approach. Bioorg. Med. Chem. Lett..

[90] Taha M., Sultan S., Imran S., Rahim F., Zaman K., Wadood A., Rehman A.U., Uddin N., Khang K.M. (2019). Synthesis of quinoline derivatives as diabetic *II* inhibitors and molecular docking studies. Bioorg. Med. Chem..

[91] George R.F., Samir E.M., Abdelhamed M.N., Abdel-Aziz H.A., Abbas S.E.S. (2019). Synthesis and anti-proliferative activity of some new quinoline based 4,5-dihydropyrazoles and their thiazole hybrids as EGFR inhibitors. Bioorg. Chem..

[92] Almandil N.B., Taha M., Rahim F., Wadood A., Imran S., Alqahtani M.A., Bamarouf Y.A., Ibrahim M., Mosaddik A., Gollapalli M. (2019). Synthesis of novel quinoline-based thiadiazole, evaluation of their antileishmanial potential and molecular docking studies. Bioorg. Chem..

[93] Yang Y., Zou W., Peng L., Yang Z., Tang Q., Chen M., Jia S., Zhang H., Lan Z., Zheng P. (2018). Design, synthesis, antiproliferative activity and docking studies of quinazoline derivatives bearing 2,3-dihydro-indole or 1,2,3,4-tetrahydroquinoline as potential EGFR inhibitors. Eur. J. Med. Chem..

[94] Zhang Y., Hou Q., Li X., Zhu J., Wang W., Li B., Zhao L., Xia H. (2019). Enrichment of novel quinazoline derivatives with high antitumor activity in mitochondria tracked by its self-fluorescence. Eur. J. Med. Chem..

[95] Li E., Lin Q., Meng Y., Zhang L., Song P., Li N., Xin J., Yang P., Bao C., Zhang D. (2019). 2,4-Disubstituted quinazolines targeting breast cancer cells via EGFR-PI3K. Eur. J. Med. Chem..

[96] El-Azab A., Abdel-Aziz A.A.M., Bua S., Nocentini A., El-Gendy M.A., Mohamed M.A., Shawer T.Z., AlSaif N.A., Supuran C.T. (2019). Synthesis of benzensulfonamides linked to quinazoline scaffolds as novel carbonic anhydrase inhibitors. Bioorg. Chem..

[97] Song J., Jang S., Lee J.W., Jung D., Lee S., Min K.H. (2019). Click chemistry for improvement in selectivity of quinazoline-based kinase inhibitors for mutant epidermal growth factor receptors. Bioorg. Med. Chem. Lett..

[98] Das D., Xie L., Wang J., Xu X., Zhang Z., Shi J., Le X., Hong J. (2019). Discovery of new quinazoline derivatives as irreversible dual EGFR/HER2 inhibitors and their anticancer activities—Part 1. Bioorg. Med. Chem. Lett..

[99] Joule J.A., Mills K. (2000). Heterocyclic Chemistry.

[100] Matos L.H.S., Masson F.T., Simeoni L.A., Mello M.H. (2018). Biological activity of dihydropyrimidinone (DHPM) derivatives: A systematic review. Eur. J. Med. Chem..

[101] Mao Q., Dai X., Xu G., Su Y., Zhang B., Liu D., Wang S. (2019). Design, synthesis and biological evaluation of 2-(4-alkoxy-3-cyano) phenyl-6-oxo-1,6-dihydropyrimidine-5-carboxylic acid derivatives as novel xanthine oxidase inhibitors. Eur. J. Med. Chem..

[102] Sanaa S., Tokalaa R., Bajaj D.M., Nagesh N., Bokarad K.K., Kiranmai G., Lakshmi U.J., Vadlamani S., Tall V., Shankaraiaha N. (2019). Design and synthesis of substituted dihydropyrimidinone derivatives as cytotoxic and tubulin polymerization inhibitors. Bioorg. Chem..

[103] Wang R., Yu S., Zhao X., Chen Y., Yang B., Wu T., Hao C., Zhao D., Cheng M. (2020). Design, synthesis, biological evaluation and molecular docking study of novel thieno[3,2-*d*]pyrimidine derivatives as potent FAK inhibitors. Eur. J. Med. Chem..

[104] Shu L., Chen C., Huan X., Huang H., Wang M., Zhang J., Yan Y., Liu J., Zhang T., Zhang D. (2020). Design, synthesis, and pharmacological evaluation of 4- or 6-phenylpyrimidine derivatives as novel and selective Janus kinase 3 inhibitors. Eur. J. Med. Chem..

[105] Diao P.C., Lin W.Y., Jian X.E., Li Y.H., You W.W., Zhao P.L. (2019). Discovery of novel pyrimidine-based benzothiazole derivatives as potent cyclin-dependent kinase 2 inhibitors with anticancer activity. Eur. J. Med. Chem..

[106] Modi P., Patel S., Chhabria M. (2019). Structure-based design, synthesis and biological evaluation of a newer series of pyrazolo[1,5-*a*]pyrimidine analogues as potential anti-tubercular agents. Bioorg. Chem..

[107] Yang F., Yu L.Z., Diao P.C., Jian X.E., Zhou M.F., Jiang C.S., You W.W., Wei-Feng M., Zhao P.L. (2019). Novel [1,2,4]triazolo[1,5-*a*]pyrimidine derivatives as potent antitubulin agents: Design, multicomponent synthesis and antiproliferative activities. Bioorg. Chem..

[108] Ma Y.Z., Tang Z.B., Sang C.Y., Qi Z.Y., Hui L., Chen S.W. (2019). Synthesis and biological evaluation of nitroxide labeled pyrimidines as Aurora kinase inhibitors. Bioorg. Med. Chem. Lett..

